# Stage-associated remodeling of CD169 macrophage states is linked to immune regulation in colitis-associated colorectal cancer

**DOI:** 10.1007/s00535-026-02463-9

**Published:** 2026-06-24

**Authors:** Yuan Xia, Yu Liu, Ai Peng, Yating Su, Rongyin Sun, Masota Tanaka, Chunhong Qiu

**Affiliations:** 1 Department of Hematology, Qilu Hospital of Shandong University, Cheeloo College of Medicine, Shandong University, Jinan, 250012 China; 2 Department of Cell Biology, School of Basic Medical Science, Cheeloo College of Medicine, Shandong University, Jinan, Shandong China; 3 State Key Laboratory of Molecular Oncology, National Cancer Center/National Clinical Research Center for Cancer/Cancer Hospital, Chinese Academy of Medical Sciences, and Peking Union Medical College, Beijing, China; 4 Department of Immunology, National Cancer Center/National Clinical Research Center for Cancer/Cancer Hospital, Chinese Academy of Medical Sciences, and Peking Union Medical College, Beijing, China; 5 Laboratory of Immune Regulation, School of Life Sciences, Tokyo University of Pharmacy and Life Science, Tokyo, Japan

**Keywords:** Colitis-associated colorectal cancer, CD169 macrophages, Macrophage heterogeneity, Macrophage heterogeneity, Tumor microenvironment

## Abstract

**Background:**

Colitis-associated colorectal cancer (CAC) is driven by chronic inflammation and immune dysregulation. However, how macrophage state heterogeneity is organized across different stages of CAC progression remains unclear.

**Methods:**

Here, using genetic depletion, single-cell transcriptomics, and pharmacological intervention, and analysis of human colitis-associated colorectal cancer specimens, we identify stage-associated remodeling of CD169-associated macrophage states during CAC progression.

**Results:**

We demonstrate that CD169 marks functionally distinct macrophage states with opposing roles: CD169-low macrophages predominate during colitis and exhibit pro-inflammatory features, whereas CD169-mid/high macrophages emerge at tumor stages, localize preferentially to para-tumor regions, and display immunosuppressive and immune-inert interaction profiles. These observations support stage-associated remodeling of CD169-associated macrophage states during CAC progression. Importantly, CD169 depletion restrains tumor growth and enhances intratumoral T cell infiltration, highlighting CD169-associated macrophages as potential therapeutic targets in inflammation-driven colorectal tumorigenesis.

**Conclusions:**

Together, our findings reveal stage-associated remodeling of CD169-associated macrophage states during CAC progression, with distinct CD169 states exhibiting divergent immune regulatory features at different disease stages. These findings highlight the importance of considering macrophage state heterogeneity when targeting CD169-associated pathways and support further investigation of state-informed therapeutic strategies in inflammation-driven colorectal tumorigenesis.

**Supplementary Information:**

The online version contains supplementary material available at https://doi.org/10.1007/s00535-026-02463-9.

## Introduction

Colitis-associated colorectal cancer (CAC) represents a prototypical malignancy driven by chronic inflammation, in which sustained immune activation and tissue damage promote malignant transformation of the intestinal epithelium [1, 2]. Unlike sporadic colorectal cancer, CAC develops through a stepwise process characterized by recurrent inflammation [3], dysregulated immune responses [4], and progressive remodeling of the tumor microenvironment [5, 6]. Although advances in immunotherapy have improved outcomes in subsets of colorectal cancer patients, effective strategies for inflammation-driven colorectal tumorigenesis remain limited, underscoring the need to better understand immune regulatory mechanisms operating during CAC progression.

Macrophages constitute a major immune component of the intestinal microenvironment and display remarkable functional plasticity in response to inflammatory cues [7–9]. During intestinal inflammation and tumor development, macrophages adopt diverse transcriptional and functional states that influence epithelial integrity, immune surveillance, and tumor growth [10–12]. Traditionally, macrophage activation has been conceptualized along the M1–M2 polarization axis; however, accumulating evidence from single-cell transcriptomic studies suggests that tumor-associated macrophages comprise multiple distinct states that cannot be fully captured by this binary framework [13, 14]. Identifying macrophage states associated with stage-dependent immune remodeling during CAC progression remains a central challenge in CAC biology.

CD169 (also known as Siglec-1) is a sialic acid-binding lectin selectively expressed by specialized macrophage populations [15–17]. CD169⁺ macrophages have been implicated in antigen capture [18], immune regulation [19], and inflammatory responses across diverse tissues [20]. In intestinal inflammation, CD169⁺ macrophages have been reported to contribute to cytokine production and immune cell recruitment, while clinical studies in colorectal cancer have yielded conflicting associations between CD169 expression and patient prognosis [21, 22]. These observations suggest that CD169⁺ macrophages represent a heterogeneous population whose roles may vary across inflammatory and tumor. However, the temporal emergence, spatial distribution, and functional properties of CD169-expressing macrophage states during CAC development remain poorly defined.

In this study, we integrated genetic depletion, pharmacological intervention, single-cell transcriptomic profiling, and in vitro functional validation to investigate how CD169-associated macrophage states evolve during CAC development. By analyzing immune cells across defined stages of disease progression, we identified stage-associated remodeling of CD169-expressing macrophage states, characterized by the increased representation of a transcriptionally distinct CD169-high population during late-stage CAC that was preferentially enriched in para-tumor regions. Our analyses further revealed that CD169 marks macrophage states with distinct immune properties across disease stages, helping explain previous observations linking CD169 to pro-inflammatory responses in colitis while also associating it with tumor progression in colorectal cancer. Mechanistically, we demonstrate that CD169⁺ macrophages may promote immunosuppressive macrophage reprogramming through APP–CD74 signaling. Finally, we show that pharmacological targeting of CD169 using the plant-derived compound cyanidin-3-O-glucoside (C3G) effectively restrains CAC progression and reshapes the immune microenvironment, highlighting CD169-associated macrophage states as potential therapeutic targets in inflammation-driven colorectal tumorigenesis.

## Results

### CD169⁺ macrophage depletion attenuates inflammation-driven colorectal tumorigenesis

**Fig. 1:**
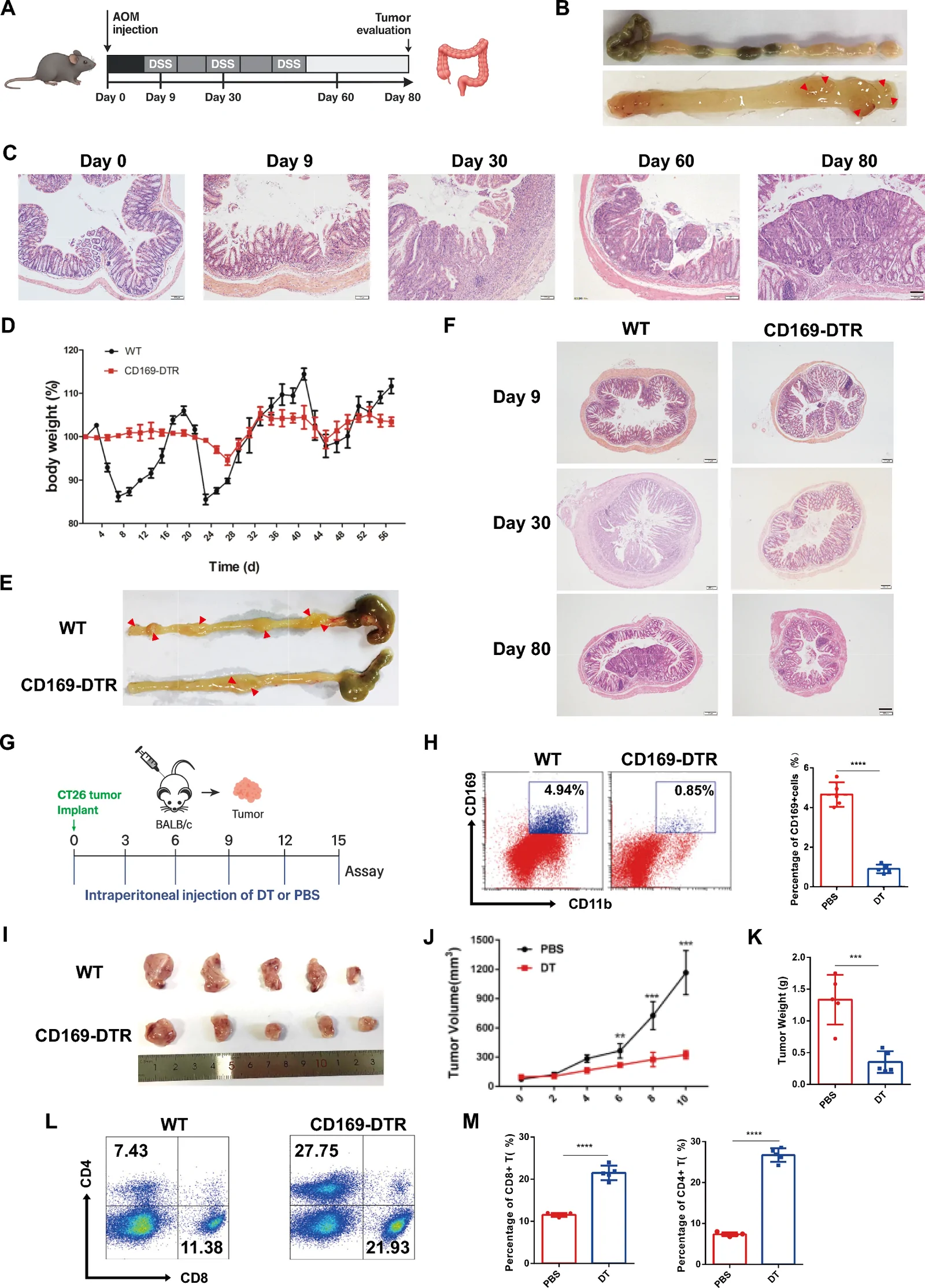
Depletion of CD169⁺ macrophages suppresses inflammation-associated colorectal tumorigenesis and enhances anti-tumor immunity. **A** Schematic illustration of the AOM/DSS-induced colitis-associated colorectal cancer (CAC) model, including DSS administration cycles and experimental endpoints. **B** Representative macroscopic images of colons from control and AOM/DSS-treated mice. Red arrowheads indicate tumor nodules. **C** Representative H&E-stained sections of colonic tissues at indicated time points during CAC progression (day 0, 9, 30, 60, and 80). Scale bar, 100 μm. **D** Body weight changes of WT and CD169-DTR mice during AOM/DSS treatment. Data are presented as mean ± SD. **E** Representative gross images of colons from WT and CD169-DTR mice at the endpoint of CAC induction. **F** Representative H&E-stained cross-sections of colons from WT and CD169-DTR mice at day 9, day 30, and day 80. Scale bar, 200 μm. **G** Experimental design of the CT26 subcutaneous tumor model with DT or PBS administration. **H** Flow cytometric analysis of CD11b⁺CD169⁺ macrophages in tumors from WT and CD169-DTR mice. **I** Representative images of excised tumors from WT and CD169-DTR mice. **J** Tumor growth curves of CT26 tumors in WT and CD169-DTR mice. **K** Tumor weights at the experimental endpoint. **L** Representative flow cytometry plots showing CD4⁺ and CD8⁺ T cells in tumors from WT and CD169-DTR mice. **M** Quantification of CD4⁺ and CD8⁺ T cell frequencies in tumors. Data are shown as mean ± SD. *P < 0.05, **P < 0.01, ***P < 0.001, ****P < 0.0001 (unpaired Student’s t-test)

To investigate the role of CD169⁺ macrophages during inflammation-associated colorectal tumorigenesis, we established a colitis-associated colorectal cancer (CAC) model [23, 24] in mice using azoxymethane (AOM) combined with dextran sulfate sodium (DSS) (Fig. 1A). Macroscopic examination of colons at the endpoint revealed extensive tumor formation in AOM/DSS-treated wild-type (WT) mice, with multiple visible tumor nodules along the colon, confirming successful model induction (Fig. 1B). Histopathological analysis further demonstrated a progressive pathological transition during CAC development. Hematoxylin and eosin (H&E) staining showed intact colonic architecture at day 0, followed by severe inflammatory infiltration and mucosal damage at day 9, dysplastic changes at day 30, and the formation of invasive adenocarcinoma at later stages (day 60 and day 80), indicating a stepwise evolution from inflammation to malignancy (Fig. 1C). WT mice and CD169-DTR mice were subjected to AOM/DSS treatment, with diphtheria toxin (DT) administration used to selectively deplete CD169⁺ macrophages. We performed absolute quantification analyses to support the selective depletion of the CD169⁺ compartment. Total CD45⁺CD11b⁺ cell numbers normalized to colon length were only mildly reduced after DT treatment, whereas absolute CD169⁺ cell counts showed a markedly greater decrease (Figure S1B). During CAC induction, WT mice exhibited a characteristic fluctuating and progressive body weight loss associated with DSS-induced colitis, whereas CD169-DTR mice showed a markedly attenuated weight loss and more rapid recovery (Fig. 1D), suggesting alleviated inflammatory injury upon CD169⁺ macrophage depletion. Consistent with this observation, macroscopic analysis revealed a significantly reduced number of colonic tumors in CD169-DTR mice compared with WT controls at the endpoint (Fig. 1E). Histological examination further confirmed that CD169⁺ macrophage depletion markedly ameliorated colonic pathology, with reduced inflammatory infiltration, preserved tissue architecture at early time points, and substantially diminished tumor burden at later stages (Fig. 1F). We additionally employed a CT26 subcutaneous tumor model as a complementary non-colitic system to determine whether the immune regulatory effects of CD169-associated macrophages extend beyond inflammation-driven CAC progression (Fig. 1G). Flow cytometric analysis of CT26 tumor tissues, using sequential gating of singlets, live cells, CD45⁺ immune cells, CD11b⁺ myeloid cells, and CD169⁺ cells (gating strategy shown in Figure S1A), demonstrated that DT treatment reduced tumor-infiltrating CD11b⁺CD169⁺ cells from 4.94% in WT controls to 0.85% in CD169-DTR mice, corresponding to an approximately 83% reduction (Fig. 1H). We further performed absolute quantification analyses. In the CT26 model, total CD45⁺CD11b⁺ myeloid cell numbers were modestly reduced following DT treatment, whereas absolute CD169⁺ cell numbers showed a substantially greater decrease (Figure S1B). With this complementary tumor model, we observed that tumors formed in CD169-DTR mice were smaller than those in WT controls (Fig. 1I), with reduced tumor growth (Fig. 1J) and lower tumor weights (Fig. 1K). Notably, depletion of CD169⁺ macrophages was accompanied by increased intratumoral T cell infiltration. Flow cytometric analysis showed elevated frequencies of both CD4⁺ and CD8⁺ T cells in tumors from CD169-DTR mice compared with WT mice (Fig. 1L, M). These findings suggest that although the CT26 model does not recapitulate CAC pathogenesis, it provides complementary evidence that CD169-associated macrophages may also contribute to tumor immune regulation in a non-colitic setting.

### Single-cell immune landscape of CAC identifies macrophage-restricted CD169 expression

**Fig. 2:**
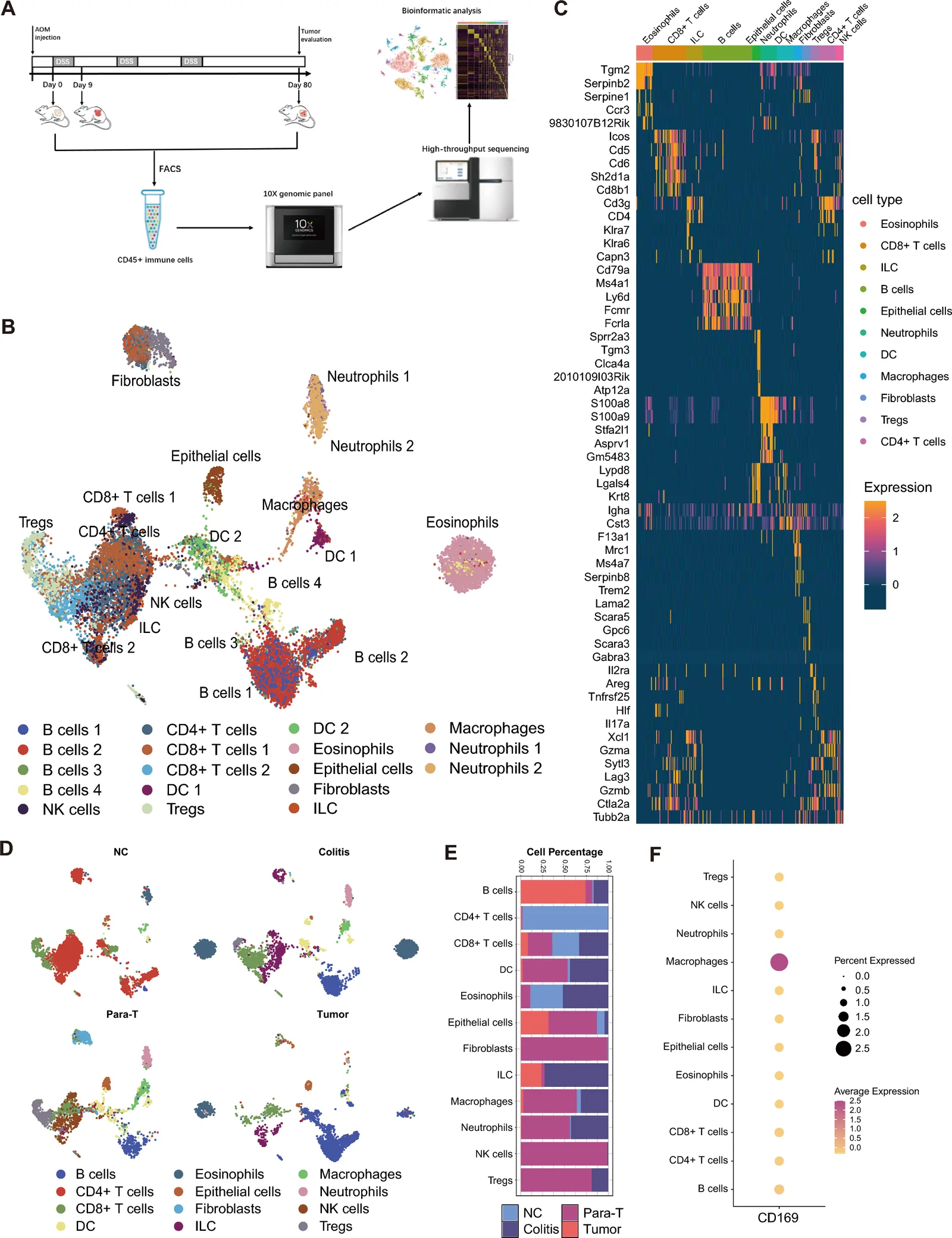
Single-cell transcriptomic landscape of immune cells during colitis-associated colorectal cancer progression. **A** Schematic overview of the experimental workflow for single-cell RNA sequencing. CD45⁺ immune cells were isolated by FACS from colonic tissues at different disease stages and subjected to 10 × Genomics-based scRNA-seq followed by bioinformatic analysis. **B** UMAP visualization of all single cells colored by annotated cell types, revealing major immune cell populations **C** Heatmap showing the expression of representative marker genes used to define each cell population. **D** UMAP projections of single cells from normal colon (NC), colitis, adjacent non-tumor tissue (Para-T), and tumor samples displayed separately. **E** Relative proportions of major cell types across different disease stages. **F** Dot plot showing the expression level and percentage of *Siglec1* (CD169) across identified cell populations, highlighting predominant enrichment in macrophages

Given that depletion of CD169⁺ macrophages markedly suppressed inflammation-driven colorectal tumorigenesis and was accompanied by enhanced intratumoral T cell responses (Fig. 1), we next sought to characterize CD169-expressing immune populations at single-cell resolution during CAC progression. To this end, we performed single-cell RNA sequencing (scRNA-seq) on fluorescence-activated cell sorting (FACS)-purified CD45⁺ immune cells isolated from colonic tissues across distinct disease stages, including normal colon (NC), colitis, adjacent non-tumor tissue (Para-T), and tumor tissue (Fig. 2A). After quality control and batch correction, unsupervised clustering analysis identified a diverse immune ecosystem comprising multiple myeloid and lymphoid populations, including macrophages, dendritic cells (DCs), neutrophils, B cells, CD4⁺ T cells, CD8⁺ T cells, regulatory T cells (Tregs), natural killer (NK) cells, innate lymphoid cells (ILCs), eosinophils, as well as non-immune stromal and epithelial populations retained during tissue processing (Fig. 2B). These clusters were annotated based on canonical marker gene expression and transcriptional signatures (Fig. 2C). When visualized separately by disease stage, immune cells from NC, colitis, Para-T, and tumor tissues displayed marked differences in cellular composition and spatial distribution across the low-dimensional embedding, indicating substantial remodeling of the immune landscape during CAC progression (Fig. 2D). Quantitative analysis of cell-type proportions revealed dynamic and stage-specific changes in immune composition. In particular, inflammatory conditions were associated with increased myeloid and innate immune cell representation, whereas tumor tissues exhibited altered distributions of lymphoid populations, consistent with an evolving tumor-associated immune environment (Fig. 2E). We next examined the expression pattern of *Siglec1* (CD169) across all identified cell types. Notably, CD169 expression was predominantly enriched in macrophages, with minimal expression detected in other immune or stromal populations (Fig. 2F). This restricted expression pattern supports the notion that CD169 marks a specific macrophage-associated transcriptional state within the CAC immune microenvironment and provides a rationale for subsequent focused analyses of CD169⁺ macrophage subsets.

### Single-cell dissection identifies a transcriptionally distinct CD169-high macrophage subset

**Fig. 3:**
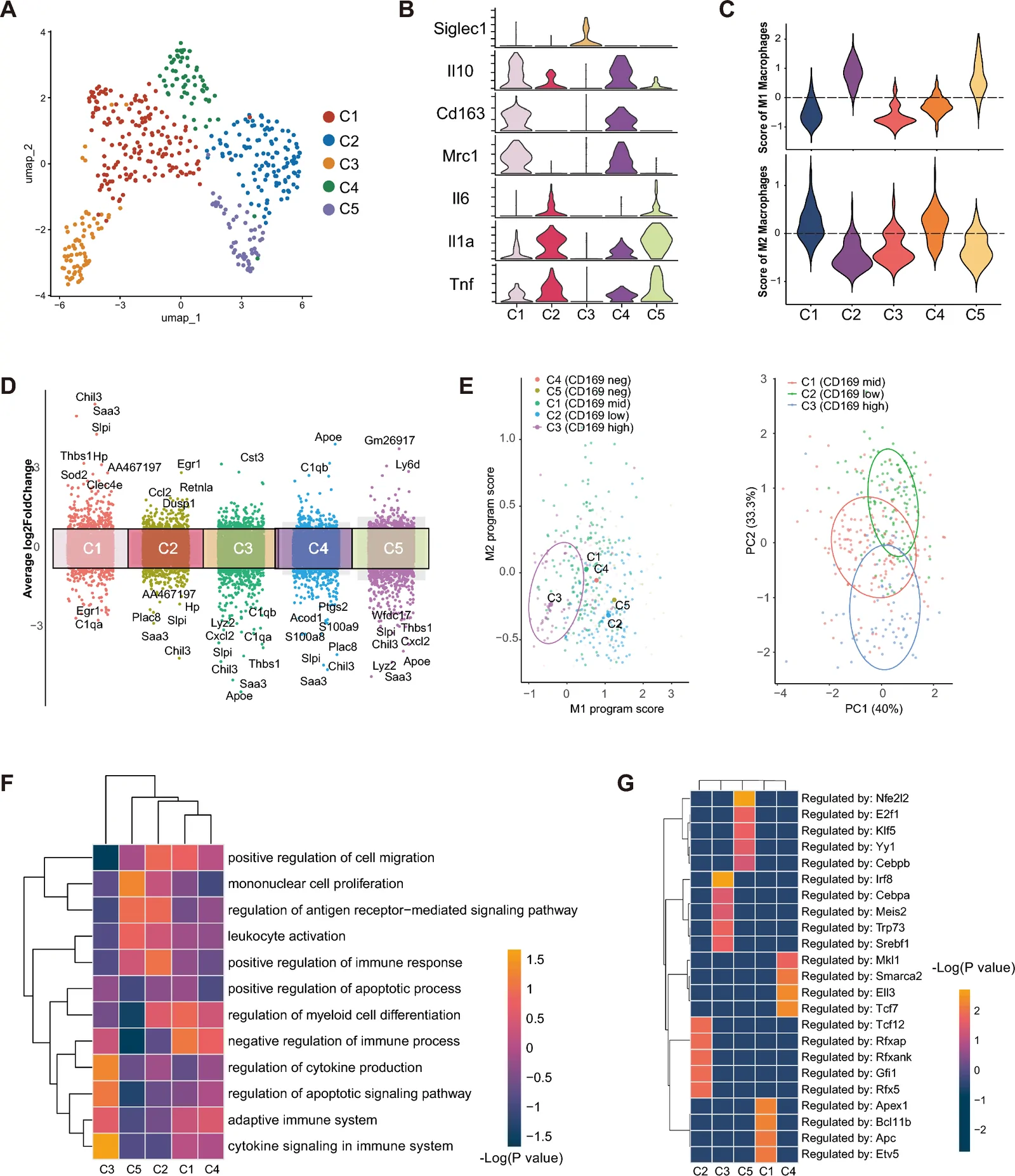
Identification of a transcriptionally distinct CD169-high macrophage subset by single-cell analysis. **A** UMAP visualization of reclustered macrophages extracted from the integrated single-cell dataset, revealing five distinct macrophage subsets (C1–C5).** B** Violin plots showing expression of *Siglec1* (CD169) and representative M1- and M2-associated markers across macrophage subsets (see also Figure S1A). **C** Scoring of M1 and M2 gene programs across macrophage subsets. **D** Heatmap showing differentially expressed genes across the five macrophage subsets. **E** Principal component analysis (PCA) depicting transcriptional relationships among macrophage subsets. Right panels show focused comparison of CD169-high, CD169-mid, and CD169-low macrophage subsets (see also Figure S1B). **F** Gene ontology enrichment analysis of differentially expressed genes for each macrophage subset. **G** Predicted upstream regulators associated with subset-specific transcriptional programs. See also Figure S1

To further resolve macrophage heterogeneity underlying CD169-associated phenotypes, we extracted macrophages from the integrated single-cell dataset and performed unsupervised reclustering analysis (Fig. 3A). This approach identified five transcriptionally distinct macrophage subsets (C1–C5), a classification that was consistently reproduced across independent analyses. We next examined the distribution of *Siglec1* (CD169) expression across these macrophage subsets, together with canonical M1- and M2-associated marker genes and program scores (Fig. 3B). Based on CD169 expression levels, the five subsets could be broadly categorized into CD169-high (C3), CD169-mid (C1), CD169-low (C2), and CD169-neg populations (C4 and C5) (Figure S2A). When evaluated in the context of classical macrophage polarization frameworks, CD169-high C3 cells displayed neither prominent M1-like nor M2-like transcriptional features. In contrast, CD169-mid macrophages (C1) were relatively enriched for M2-associated signatures, whereas CD169-low macrophages (C2) preferentially exhibited M1-associated features. CD169-neg subsets (C4 and C5) largely aligned with conventional macrophage states, suggesting that CD169 expression delineates macrophage states beyond the traditional M1/M2 dichotomy. Consistent with these observations, differential gene expression analysis revealed substantial transcriptional divergence among the five macrophage subsets (Fig. 3D). In particular, C3 displayed a unique gene expression profile that was distinct from all other subsets. To assess the global transcriptional relationships among macrophage subsets, we performed principal component analysis (PCA). This analysis demonstrated that CD169-high C3 macrophages were clearly separated from other subsets in low-dimensional space, whereas C1 clustered closely with C4, and C2 showed strong similarity to C5 (Fig. 3E). Importantly, when analysis was restricted to CD169-expressing subsets, CD169-high macrophages again segregated distinctly from CD169-mid and CD169-low populations (Fig. 3E and Figure S2B), reinforcing the transcriptional uniqueness of the CD169-high state. Finally, functional annotation of subset-specific differentially expressed genes revealed marked differences in biological programs across macrophage subsets. Gene ontology (GO) enrichment and upstream regulator analyses indicated that CD169-high C3 macrophages were preferentially associated with pathways related to negative regulation of immune responses, in contrast to other subsets that were enriched for inflammatory or homeostatic programs (Fig. 3F, G). Independent pathway analysis focusing specifically on C3 further confirmed enrichment of immune-suppressive and immune-regulatory processes (Figure S2C). Collectively, these results identify CD169-high macrophages as a transcriptionally distinct subset within the CAC microenvironment, characterized by a unique gene expression program that is not adequately captured by classical macrophage polarization paradigms.

### Spatiotemporal features of CD169-high macrophages during CAC progression

**Fig. 4:**
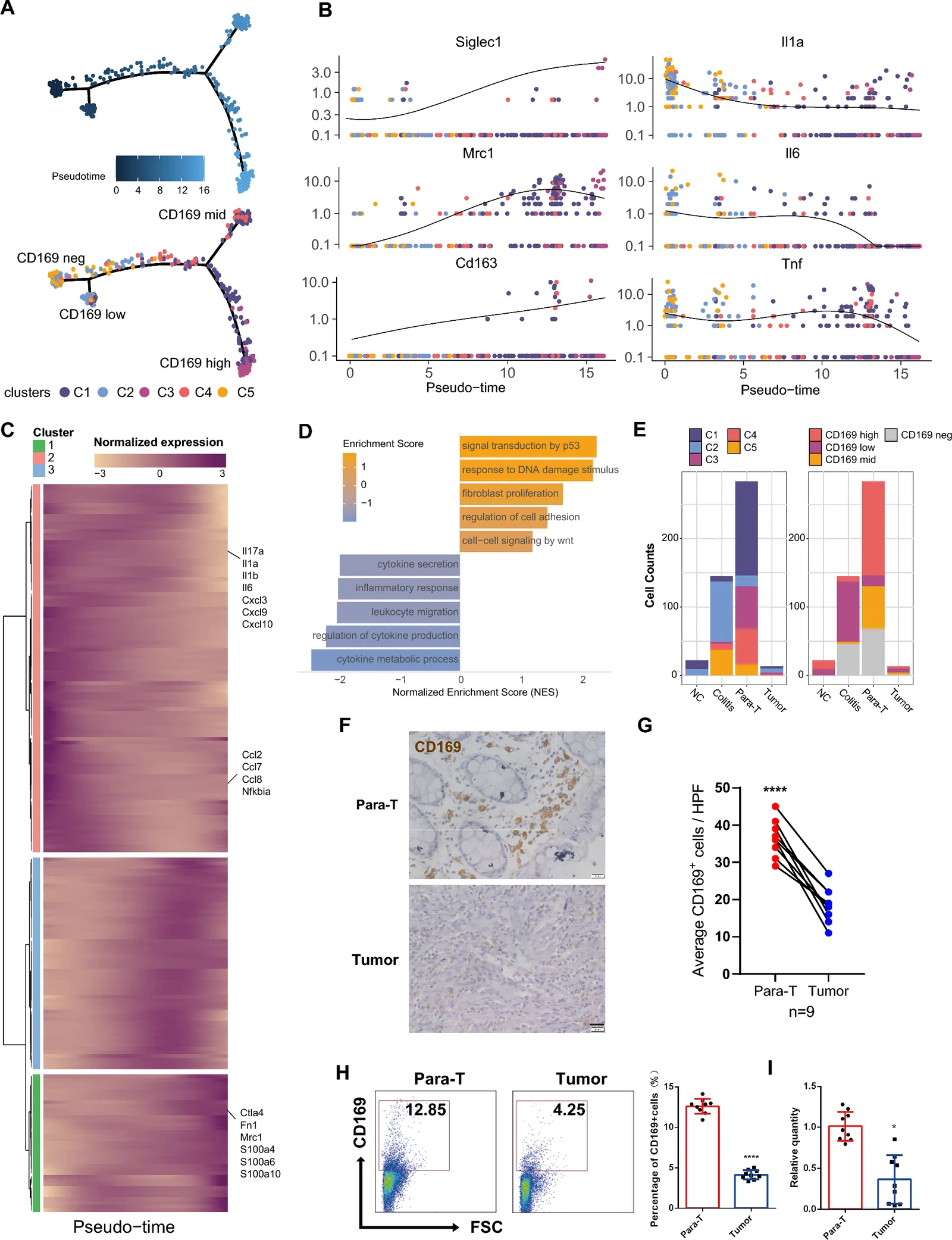
Dynamic and spatial characterization of CD169-associated macrophage states during CAC progression. **A** Pseudotime trajectory of macrophage subsets inferred from single-cell transcriptomic data, with cells colored by CD169 expression state. **B** Expression dynamics of *Siglec1* (CD169), M2-associated markers (*Mrc1*, *Cd163*), and inflammatory cytokines (*Il1a*, *Il6*, *Tnf*) along pseudotime. **C** Heatmap showing genes dynamically regulated along pseudotime, illustrating gradual transcriptional changes associated with macrophage state remodeling. **D** Gene set enrichment analysis (GSEA) of pseudotime-associated genes, highlighting pathways progressively enriched or depleted along the trajectory. **E** Distribution of macrophage subsets across disease stages (NC, colitis, para-tumor, and tumor tissues). **F** Representative immunohistochemical staining of CD169 in paired human colitis-associated colorectal cancer para-tumor and tumor tissues. Representative immunohistochemical staining of CD169 in paired human colitis-associated colorectal cancer para-tumor and tumor tissues. Para-tumor regions were defined as histologically confirmed non-tumor tissues immediately adjacent to tumor lesions based on pathological evaluation. In the tumor panel, the entire field of view represents histologically confirmed tumor tissue rather than a mixed tumor-adjacent region. Representative H&E images are provided as pathological references for tissue annotation. **G** Representative immunohistochemical staining and quantification of CD169-positive cells in paired human colitis-associated colorectal cancer para-tumor and tumor tissues (*n* = 9 patients). Para-tumor regions were defined as histologically confirmed non-tumor tissues immediately adjacent to tumor lesions based on pathological evaluation. CD169-positive cells were manually counted across two sections per sample and five high-power fields per section. Data were analyzed using paired Student’s t-test. **H** Flow cytometric analysis of CD169⁺ cells in paired freshly isolated human colitis-associated colorectal cancer tumor and para-tumor tissues (*n* = 9 patients). Representative flow plots and quantification are shown **I** Quantitative PCR analysis of Siglec1 expression in paired human colitis-associated colorectal cancer tumor and para-tumor tissues from 9 patients. Data are shown as mean ± SD. *P < 0.05, **P < 0.01, ***P < 0.001

To further explore potential relationships among macrophage subsets, we performed pseudotime trajectory analysis on reclustered macrophages using Monocle v2. Pseudotime analysis computationally orders cells based on transcriptional similarity and is commonly used to infer potential relationships among cellular states, although it does not provide direct evidence of lineage relationships. This analysis revealed a continuous transcriptional continuum encompassing CD169-low, CD169-mid, and CD169-high macrophage states (Fig. 4A). CD169-low macrophages were predominantly positioned at early pseudotime stages, whereas CD169-high macrophages were enriched at later pseudotime stages, suggesting a progressive transcriptional shift toward a CD169-high state during CAC progression. Overlaying macrophage polarization features onto the pseudotime trajectory showed that early-stage macrophages were enriched for M1-associated characteristics, consistent with an inflammatory state likely corresponding to colitis. As pseudotime progressed, macrophages gradually exhibited increased expression of M2-associated markers and were associated with an intermediate CD169-mid state, ultimately converging toward CD169-high-associated transcriptional programs at later pseudotime stages (Fig. 4A). These observations suggest that CD169-high macrophages are preferentially associated with later stages of inflammation-driven tumor progression. Consistent with this trajectory, expression dynamics of key marker genes along pseudotime further supported a coordinated transcriptional shift *Siglec1* (CD169) expression progressively increased over pseudotime, accompanied by a gradual upregulation of M2-associated genes (*Mrc1*, *Cd163*), whereas inflammatory cytokines and M1-associated genes (*Il1a*, *Il6*, *Tnf*) showed an overall decreasing trend (Fig. 4B).

To systematically characterize transcriptional changes associated with stage-associated remodeling of macrophage states, we examined genes dynamically regulated along pseudotime. Heatmap visualization revealed gradual attenuation of inflammatory gene expression and a concomitant increase in genes associated with immune regulation and tissue remodeling as macrophages progressed along pseudotime (Fig. 4C). Gene set enrichment analysis (GSEA) of pseudotime-associated genes further demonstrated that pathways related to inflammatory responses, cytokine production, and immune activation were progressively downregulated over pseudotime. In contrast, pathways associated with DNA damage responses, cell adhesion, fibroblast proliferation, and Wnt signaling exhibited increasing enrichment at later pseudotime stages (Fig. 4D), consistent with tumor-associated transcriptional programs.

We next examined the distribution of macrophage subsets across disease stages. Quantitative analysis revealed that colitis samples were dominated by CD169-low macrophages, whereas tumor tissues were largely enriched for CD169-mid and CD169-high populations (Fig. 4E). Notably, both CD169-mid and CD169-high macrophages were preferentially detected in para-tumor regions rather than within tumor cores, suggesting a spatially restricted distribution. To validate these observations in human colitis-associated colorectal cancer (CAC) specimens, immunohistochemical staining of paired patient samples confirmed elevated CD169 expression in para-tumor regions compared with tumor tissues (Fig. 4F). Quantitative analysis further confirmed significantly increased CD169⁺ cell abundance in para-tumor tissues relative to paired tumor samples (Fig. 4G). Para-tumor tissues were defined as histologically confirmed non-tumor tissues immediately adjacent to tumor lesions based on pathological evaluation, and representative H&E images (Figure S3A). Consistently, flow cytometric analysis of additional CAC patient samples revealed a higher frequency of CD169⁺ cells in para-tumor tissues than in tumor tissues (Fig. 4H, gating strategy shown in Figure S3B), which was further supported by quantitative PCR analysis showing reduced SIGLEC1 expression in tumor tissues (Fig. 4I). Although these findings support enrichment of CD169⁺ cells in para-tumor regions, single-marker CD169 immunohistochemistry does not directly resolve the transcriptionally distinct CD169-low, CD169-mid, and CD169-high macrophage states identified by single-cell RNA sequencing. As additional supportive evidence for broader colorectal cancer relevance, analysis of TCGA colorectal cancer cohorts, which predominantly represent sporadic CRC, showed that SIGLEC1 expression was higher in para-tumor tissues than in tumor tissues in both colon adenocarcinoma (COAD) and rectal adenocarcinoma (READ) datasets (Figure S3C). Together, these analyses demonstrate that CD169-associated macrophages comprise transcriptionally distinct states that are temporally and spatially segregated during CAC progression, with inflammatory CD169-low states predominating during colitis and immunosuppressive CD169-mid/high states preferentially enriched in para-tumor regions.

### CD169-mid/high macrophages display an immunosuppressive interaction state

**Fig. 5:**
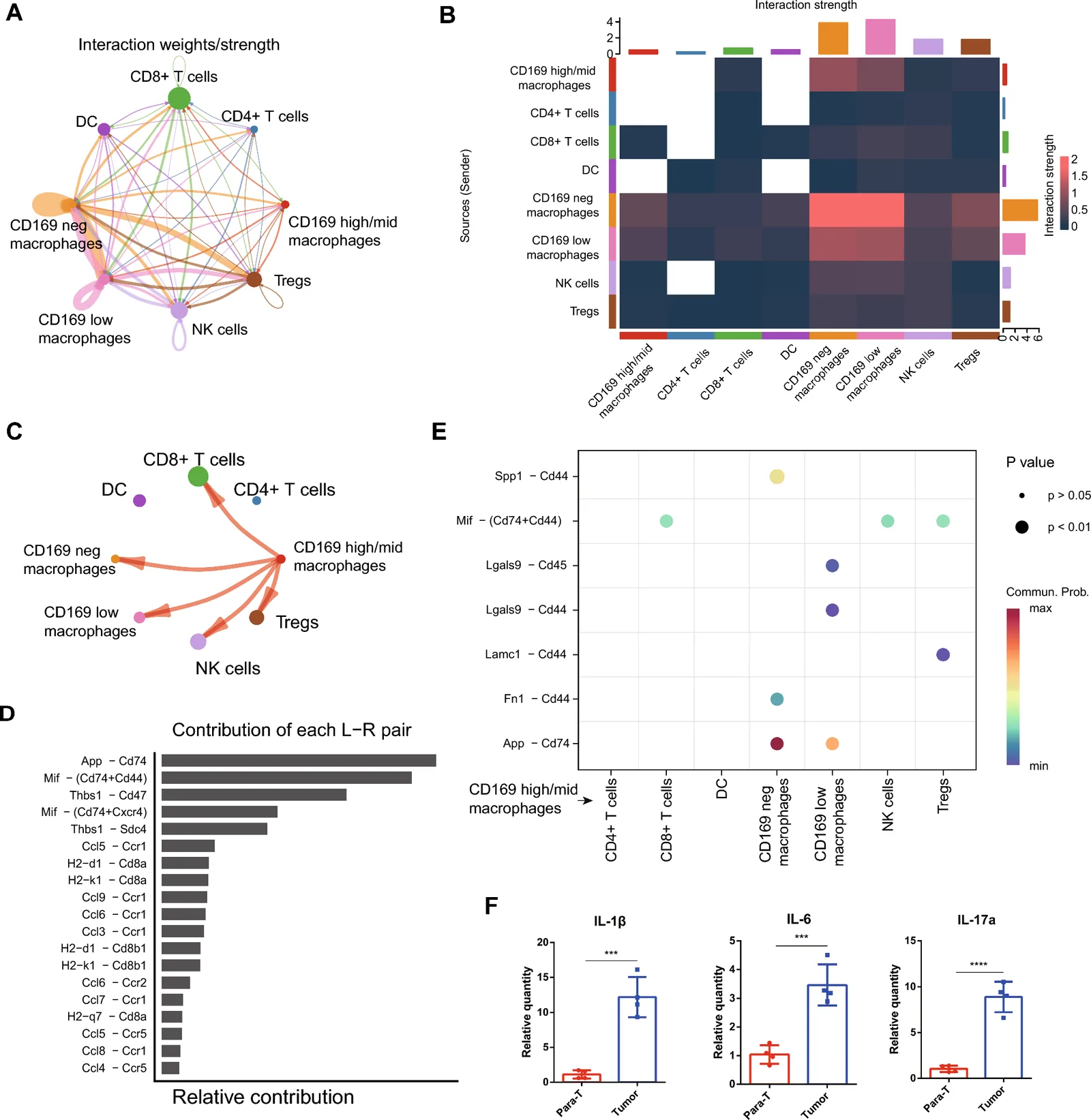
Cell–cell communication analysis reveals immunoregulatory interaction patterns associated with CD169-mid/high macrophages. **A** Global overview of cell–cell communication strength among macrophage subsets and other immune populations. **B** Relative interaction intensity between CD169-mid/high macrophages and other macrophage subsets. **C** Network representation of ligand–receptor interactions originating from CD169-mid/high macrophages. **D** Ranking of dominant ligand–receptor pairs within CD169-mid/high macrophage-associated communication networks, highlighting APP–CD74 signaling. **E** Predicted interactions between CD169-mid/high macrophages and other immune cells, emphasizing APP–CD74- and SPP1–CD44-associated signaling pathways. **F** Comparative analysis of inflammatory cytokine expression between para-tumor and tumor tissues. Data are shown as mean ± SD. Statistical significance was assessed using unpaired Student’s *t*-test where applicable

Given that functional annotation of CD169-high macrophages indicated enrichment of immunoregulatory programs, we next sought to explore how these macrophage populations interact with other immune cells within the CAC microenvironment. Although CD169-mid and CD169-high macrophages displayed substantial transcriptional heterogeneity in Fig. 3, many of these differences reflected broader functional diversity beyond immune regulation. Notably, both populations consistently shared immunosuppressive characteristics, including enrichment of immunoregulatory pathways and preferential localization within paratumoral regions. Therefore, to specifically investigate their shared immunosuppressive interactions within the CAC microenvironment, CD169-mid and CD169-high macrophages were analyzed together as a unified population for subsequent cell–cell communication analysis. Global assessment of ligand–receptor interactions revealed that, compared with other macrophage subsets, CD169-mid/high macrophages exhibited a relatively limited overall interaction strength with surrounding immune cells (Fig. 5A), suggesting a low-communication or immune-inert interaction profile. Despite this globally reduced interaction landscape, CD169-mid/high macrophages displayed preferential communication with CD169-low and CD169-neg macrophage populations (Fig. 5B). To further delineate these interactions, we focused specifically on signaling networks originating from CD169-mid/high macrophages. Network analysis revealed that APP–CD74 represented one of the most prominent ligand–receptor pairs within this interaction module (Fig. 5C, D). We next examined the downstream targets of CD169-mid/high macrophage signaling across immune cell populations. Notably, CD169-mid/high macrophages were predicted to preferentially engage other macrophage subsets through APP–CD74 signaling, a pathway linked to immune tolerance and suppression. In parallel, enrichment of SPP1–CD44 interactions, commonly associated with exhausted and suppressive myeloid states, was observed, suggesting that CD169-mid/high macrophages may contribute to an immunoregulatory microenvironment characterized by macrophage suppression and exhaustion rather than immune activation (Fig. 5E). To contextualize these observations within the tissue environment, we compared inflammatory gene expression between paratumor and tumor tissues. Analysis revealed that paratumor regions exhibited markedly lower expression of inflammatory cytokines compared with tumor tissues (Fig. 5F). Finally, to further validate the suppressive function of these CD169⁺ macrophages in vitro, we isolated CD11b⁺CD169⁺ and CD11b⁺CD169⁻ macrophages from paratumoral colon tissues of CAC mice and established a transwell co-culture system (Figure S4A; gating strategy shown in Figure S4B). We focused on paratumoral tissues because our single-cell analysis showed that CD169⁺ macrophages in these regions were primarily enriched for the immunosuppressive CD169-mid and CD169-high subsets. Consistent with our cell–cell communication analysis, CD169⁺ macrophages exhibited significantly higher App expression, whereas CD169⁻ macrophages showed relatively elevated Cd74 expression (Figure S4C). we found that CD169⁺ macrophages induced the expression of immunosuppressive genes, including Tgfb1, Arg1, and Il10, in CD169⁻ macrophages. Notably, blockade of APP or CD74 largely reversed these effects (Figure S4D), suggesting that APP–CD74 signaling may contribute to the acquisition of immunoregulatory features in macrophages.

### C3G reduces CD169 expression and reshapes the CAC immune microenvironment

**Fig. 6:**
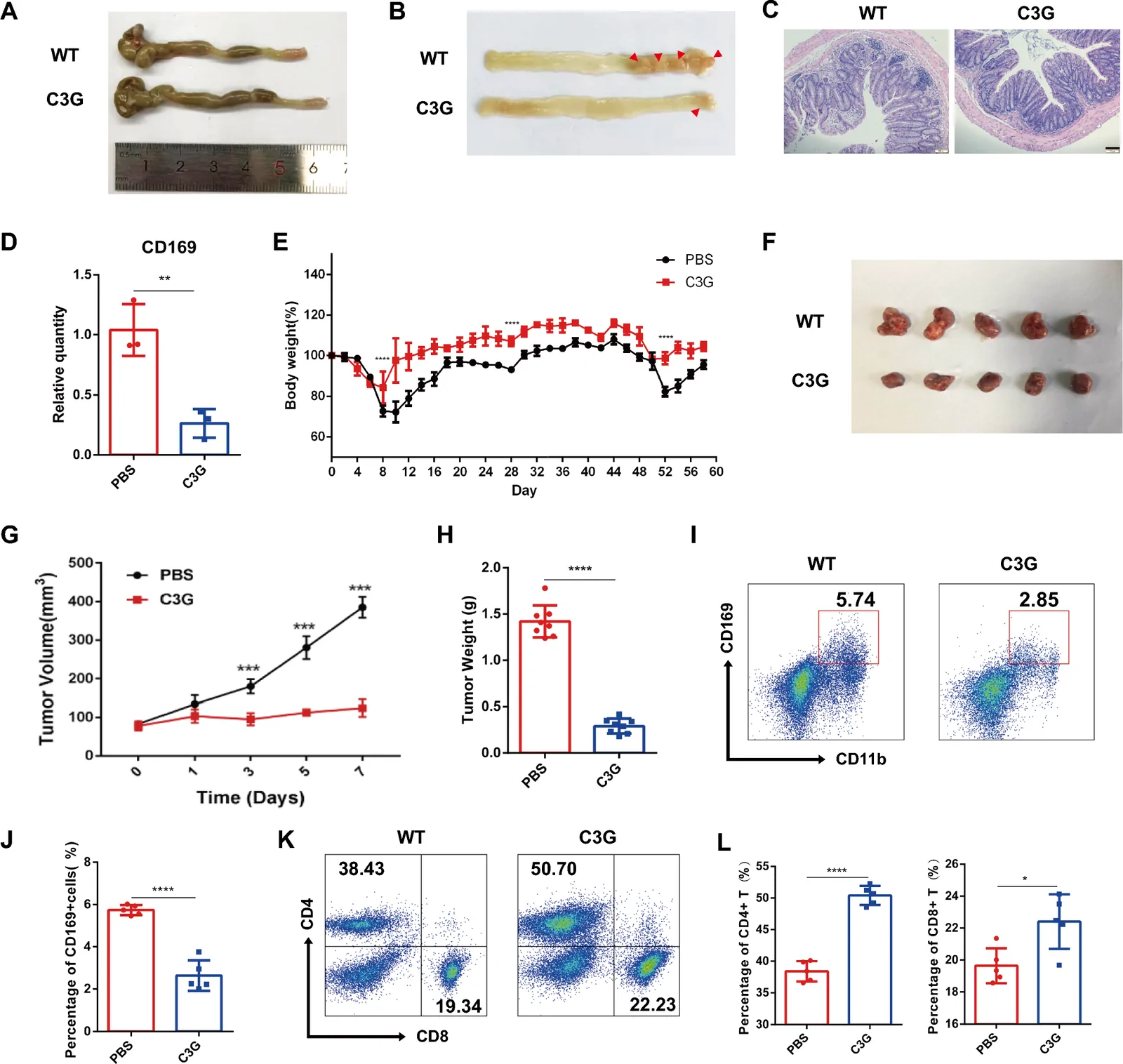
C3G reduces CD169 expression, suppresses colorectal tumor progression, and enhances anti-tumor immunity. **A** Representative macroscopic images of colons from AOM/DSS-treated mice receiving vehicle or C3G. **B** Quantification of colonic tumor numbers in control and C3G-treated mice. **C** Representative H&E-stained sections of colonic tissues from control and C3G-treated mice. **D** Quantitative analysis of CD169 expression in colonic tissues following C3G treatment. **E** Body weight changes during CAC induction in vehicle- and C3G-treated mice. **F** Representative images of CT26 subcutaneous tumors from control and C3G-treated mice. **G** Tumor growth curves in the CT26 subcutaneous model. **H** Tumor weights at the experimental endpoint. **I** Representative flow cytometry plots showing CD11b^+^CD169^+^ macrophages in tumors. **J** Quantification of CD169^+^ macrophages following C3G treatment. **K** Representative flow cytometry plots showing CD4^+^ and CD8^+^ T cells in tumors. **L** Quantification of intratumoral CD4^+^ and CD8^+^ T cells. Data are shown as mean ± SD. *P < 0.05, **P < 0.01, ***P < 0.001, ****P < 0.0001

Building on the identification of CD169-mid/high macrophages as a temporally and spatially restricted immunosuppressive state during CAC progression, we next explored whether pharmacological modulation of CD169-associated pathways could therapeutically modulate disease outcomes. Cyanidin-3-O-glucoside (C3G), a compound previously shown by us to suppress type I IFN-induced CD169 expression [25], was therefore evaluated in both inflammation-driven and tumor models. Dose optimization experiments revealed that 4 μM C3G achieved the greatest reduction in CD169 expression, whereas higher concentrations did not confer additional benefit (Figure S5A). Using this optimized dose, C3G was administered in the AOM/DSS-induced CAC model. Macroscopic examination showed that C3G-treated mice exhibited markedly elongated colons compared with vehicle-treated controls, indicative of alleviated disease severity (Fig. 6A). Consistently, the number of colonic tumors was significantly reduced following C3G treatment (Fig. 6B). Histopathological analysis further demonstrated improved colonic architecture in C3G-treated mice, with reduced epithelial dysplasia and inflammatory infiltration (Fig. 6C). Quantitative PCR analyses confirmed that C3G treatment significantly decreased CD169 expression in colonic tissues (Fig. 6D). Immunofluorescence staining of paratumor regions further validated the effective suppression of CD169 by C3G in situ (Figure S5B). To further determine whether C3G broadly affected major immune cell compartments, we performed an additional short-term validation experiment in WT mice treated with C3G. Colonic immune cells were collected after treatment and analyzed by flow cytometry using a defined gating strategy (Figure S6A). C3G treatment did not markedly alter the proportions of total CD45^+^CD11b^+^ myeloid cells, monocytes, neutrophils, dendritic cells, T cells, or B cells (Figure S6B), suggesting that C3G did not induce broad leukocyte depletion but preferentially affected CD169-associated macrophages. During the CAC induction, C3G-treated mice displayed attenuated body weight loss compared with control mice (Fig. 6E). To further determine whether the protective effects of C3G were dependent on the CD169 axis in vivo, we performed an additional epistasis experiment in DSS-treated CD169-DTR mice. Both DT-mediated CD169 depletion and C3G treatment significantly alleviated colitis severity, as reflected by increased colon length and improved body weight recovery compared with PBS-treated controls. Importantly, combined DT + C3G treatment did not confer additional therapeutic benefit compared with DT treatment alone (Figure S6C–E), supporting a potential contribution of CD169-associated pathways to the protective effects of C3G during DSS-induced colitis. However, these findings were obtained in the DSS colitis model and do not directly address whether the tumor-suppressive effects of C3G observed in the AOM/DSS-CAC model are likewise CD169-dependent.

We also employed a CT26 subcutaneous tumor model to further validate the therapeutic effect of C3G in an independent tumor setting. C3G treatment resulted in visibly smaller tumors (Fig. 6F), accompanied by significantly reduced tumor growth kinetics (Fig. 6G) and lower tumor weights at the experimental endpoint (Fig. 6H). Flow cytometric analysis confirmed a marked reduction in CD169^+^ macrophages within tumors following C3G treatment (Fig. 6I, J). Concomitantly, C3G administration was associated with a pronounced increase in intratumoral CD4^+^ and CD8^+^ T cell infiltration (Fig. 6K, L), suggesting improved antitumor immune activity that may be partially associated with reduced CD169-associated immune suppression. In addition to its immunomodulatory effects, supplementary analyses revealed that C3G exerted a modest but measurable direct antitumor effect by promoting apoptosis of tumor cells in vitro (Figure S5C–F). Together, these findings highlight C3G as a plant-derived and readily accessible compound with therapeutic potential, whose effects may involve both modulation of CD169-associated immune states and modest direct antitumor activity.

## Discussion

In this study, we demonstrate that CD169 expression delineates distinct macrophage states with divergent functional properties during colitis-associated colorectal cancer (CAC) progression. Rather than representing a uniform macrophage population, CD169 marks a spectrum of macrophage states that differ in transcriptional programs, temporal emergence, spatial distribution, and immune regulatory capacity. By integrating single-cell transcriptomics, genetic depletion, and pharmacological intervention, our work suggests that CD169-associated macrophage states contribute to distinct stages of inflammation-driven tumorigenesis. Although CD169-associated macrophages may also contribute to tumor progression in non-inflammatory settings, as suggested by the CT26 model, the state-dependent heterogeneity identified in this study, including temporal remodeling, spatial redistribution, and the emergence of immunosuppressive CD169-mid/high states, was specifically defined within the context of colitis-associated colorectal cancer.

A key finding of our study is that CD169 expression stratifies macrophages into at least three functionally distinct states, CD169-low, CD169-mid, and CD169-high, which cannot be resolved by bulk or single-marker-based analyses. Traditional approaches that assess CD169 expression at the tissue level or by conventional flow cytometry inherently collapses these heterogeneous states into a single population [26, 27], obscuring their distinct biological roles. Single-cell resolution reveals that CD169-low macrophages dominate early inflammatory stages and exhibit pro-inflammatory features, whereas CD169-mid and CD169-high macrophages are enriched at later stages of CAC and are associated with immunosuppressive and immune-inert interaction profiles. These findings underscore the importance of state-specific resolution when interpreting the functional significance of CD169 in complex disease settings. Our data further indicate that CD169-associated macrophage states extend beyond the classical M1–M2 polarization framework. While CD169-low macrophages partially align with inflammatory, M1-like characteristics and CD169-mid macrophages show enrichment of M2-associated features, CD169-high macrophages represent a transcriptionally distinct state that does not conform to either category. This observation demonstrating that tumor-associated macrophages occupy diverse functional states cannot be adequately described by binary polarization models. The identification of a CD169-high macrophage state with unique transcriptional and interaction properties highlights the need to move beyond simplified macrophage classifications, particularly in inflammation-driven cancers.

Importantly, our findings may help contextualize the seemingly contradictory roles of CD169 in intestinal disease. Previous studies have largely characterized CD169^+^ macrophages as pro-inflammatory mediators in colitis [15, 28], promoting cytokine production and immune cell recruitment [25, 29, 30]. In contrast, clinical observations in colorectal cancer have reported inconsistent associations between CD169 expression and disease outcome, without mechanistic explanation [31, 32]. Notably, a recent study by Hu et al. demonstrated that CD169⁺ macrophages can directly recognize and eliminate colorectal tumor cells, supporting an antitumor role for this population [31]. Similarly, previous studies have reported that increased CD169⁺ macrophages in regional lymph nodes are associated with favorable prognosis in colorectal cancer [33], likely reflecting antitumor immune activation. Notably, these findings do not necessarily conflict with the tumoricidal CD169⁺ macrophages reported by Hu et al. Instead, they may reflect the functional heterogeneity of CD169-associated macrophages across disease stages and tissue contexts. In our study, CD169-low macrophages were enriched during inflammatory stages and retained pro-inflammatory and immune-activating features, which may be compatible with previously reported antitumor functions of CD169⁺ macrophages. In contrast, CD169-mid and CD169-high macrophage states were preferentially enriched in paratumor regions and displayed immunosuppressive interaction programs. However, because tumor-killing activity was not directly assessed in the current study, we cannot determine whether the CD169-low state corresponds functionally to the tumoricidal CD169⁺ macrophages described by Hu et al. Defining the relationship between inflammatory/immune-activating CD169-low macrophages, immunoregulatory CD169-mid/high macrophages, and previously reported tumoricidal CD169⁺ macrophages will require further investigation. Our cell–cell communication analyses further support an association between CD169-mid/high macrophages and immunoregulatory features within the CAC microenvironment. These macrophage states exhibit limited global communication with immune cells yet preferentially engage other macrophage subsets through APP–CD74 and SPP1–CD44 signaling axes, pathways that have previously been associated with immune tolerance, macrophage exhaustion, and suppression of inflammatory activity [34, 35]. The spatial enrichment of CD169-mid/high macrophages in paratumor regions, together with their predicted communication profiles and in vitro effects on macrophage gene expression, suggests that these cells may contribute to the development of a local immunoregulatory environment. However, further studies will be required to determine whether APP–CD74 signaling directly mediates T cell suppression or promotes tumor progression in vivo.

From a translational perspective, our study identifies CD169-associated immune regulation as a potentially tractable therapeutic axis in CAC. Both genetic depletion and pharmacological intervention targeting CD169-associated pathways restrained tumor progression and enhanced intratumoral T cell infiltration, suggesting that disrupting CD169-associated macrophage states may reshape the immune landscape. Notably, the plant-derived compound cyanidin-3-O-glucoside (C3G) [36, 37] suppressed CD169 expression and produced therapeutic effects that partially overlapped with those observed in CD169-depleted models [25]. Given its natural origin, accessibility, and potential dual capacity to modulate immune states while exerting modest direct antitumor effects, C3G warrants further investigation as a potential therapeutic strategy in inflammation-driven colorectal tumorigenesis. However, although our DSS-colitis epistasis experiments support a role for the CD169 axis in mediating the protective effects of C3G during intestinal inflammation, these studies were not performed in the AOM/DSS-CAC model. Therefore, the current data do not directly establish whether the tumor-preventive effects of C3G during CAC progression are likewise CD169-dependent, and the relationship between C3G-mediated inflammation control and tumor suppression requires further investigation.

Several limitations should be acknowledged in this study. First, although our human validation analyses were performed using specimens from patients with colitis-associated colorectal cancer (CAC), these analyses were limited to comparisons between tumor and paratumor tissues collected at the time of cancer diagnosis. Therefore, our human data do not directly capture or validate the full disease continuum from ulcerative colitis-associated inflammation through dysplasia to CAC. Future studies incorporating longitudinal human specimens spanning these disease stages will be required to further validate the stage-associated remodeling of macrophage states identified in our experimental models. In addition, although TCGA COAD/READ analyses provided supportive evidence for broader colorectal cancer relevance, these cohorts predominantly represent sporadic colorectal cancer and therefore do not constitute CAC-specific validation of the mechanisms identified in this study. Furthermore, although the CT26 subcutaneous tumor model provided complementary evidence that CD169-associated macrophages may influence tumor progression beyond inflammatory settings, this model does not recapitulate colitis-driven tumorigenesis, intestinal carcinogenesis, or the colonic inflammatory microenvironment. Therefore, the CT26 findings should be interpreted as supportive observations and cannot be considered direct evidence for CAC-specific mechanisms.

Secondly, although CD169⁺ cell depletion was quantitatively validated in several key tissues and experimental settings, the anatomical scope of depletion was not comprehensively assessed across all organs and disease stages. Therefore, tissue-specific and temporal differences in depletion efficiency cannot be completely excluded. Moreover, the CD169-DTR model used in this study induces systemic depletion of Siglec1-expressing cells following DT administration rather than intestine-restricted targeting. Consequently, contributions from CD169⁺ cell populations residing outside the intestinal compartment cannot be completely excluded and may represent a potential confounding factor when interpreting the observed phenotypes. Previous studies have demonstrated systemic depletion of CD169⁺ macrophages across multiple anatomical sites following DT administration [38]. In line with these observations, our current study further validated depletion efficiency in both AOM/DSS colon tissues and CT26 tumor tissues. Nevertheless, depletion efficiency was not comprehensively quantified across all organs and disease stages examined in this study. In addition, the CD45⁺CD11b⁺ population used for absolute quantification represents a broad myeloid compartment rather than a macrophage-specific population. Therefore, these measurements should be interpreted as overall myeloid-cell abundance and do not provide a direct assessment of total macrophage numbers, although they remain useful for estimating broader changes within the myeloid compartment.

Thirdly, although short-term C3G treatment did not markedly alter major leukocyte populations in healthy mice, the immune selectivity of C3G under inflammatory or tumor-bearing conditions was not comprehensively evaluated. Because immune-cell composition and function may differ substantially during DSS colitis and CAC progression, additional disease-context immune profiling, including absolute quantification and functional characterization of immune-cell populations, will be required to fully define the selectivity of C3G in these settings. Furthermore, although C3G reduced CD169 expression and partially phenocopied the effects of CD169 depletion, the current study did not employ CD169-blocking antibodies, Siglec1 knockout/knockdown approaches, or macrophage-specific genetic manipulation. Therefore, the contribution of direct CD169 inhibition to the observed effects of C3G cannot be conclusively established. In addition, while our spatial analyses identified differential enrichment of CD169-associated macrophage states in tumor and paratumor regions, the broader tissue distribution of these populations across additional anatomical sites remains incompletely characterized. The spatial validation experiments were based on single-marker CD169 immunohistochemistry and therefore support spatial enrichment of CD169⁺ cells but do not directly distinguish the transcriptionally defined CD169-low, CD169-mid, and CD169-high macrophage states identified by single-cell analyses. Higher-resolution approaches, such as multiplex immunofluorescence, spatial transcriptomics, or state-specific marker panels, will be required to resolve these macrophage states in situ.

Finally, although our findings support functional heterogeneity among CD169-associated macrophage states and suggest that CD169-low macrophages retain inflammatory and immune-activating features, the current study did not directly assess tumoricidal activity of CD169⁺ macrophages. Therefore, we cannot determine whether the CD169-low state identified here corresponds functionally to the tumoricidal CD169⁺ macrophages reported in previous studies. Further studies will be required to define the relationship between inflammatory/immune-activating CD169-low macrophages, immunoregulatory CD169-mid/high macrophages, and previously described antitumor CD169⁺ macrophage populations. And although pseudotime analysis provided useful insight into potential transcriptional relationships among macrophage states, it infers cellular ordering based on transcriptional similarity and does not constitute direct evidence of lineage conversion. Future studies using lineage tracing and higher-resolution spatial approaches will be needed to further validate these findings. In addition, although APP–CD74 signaling was identified as a prominent communication pathway associated with CD169-associated macrophages and was supported by in vitro functional experiments, the current study did not directly determine whether this signaling axis mediates T cell suppression or promotes tumor progression in vivo. Further studies incorporating APP/CD74 blockade, T cell functional analyses, and tumor rescue experiments will be required to define the mechanistic contribution of APP–CD74 signaling during CAC progression.

In summary, our work redefines CD169 not as a static macrophage marker, but as an indicator of dynamic macrophage states with context-dependent functions during inflammation-driven colorectal tumorigenesis. By resolving the apparent contradiction between the pro-inflammatory role of CD169 in colitis and its association with tumor progression, this study provides a framework for understanding macrophage plasticity in CAC and highlights CD169-associated macrophage states as potential targets for future therapeutic investigation.

## Materials and methods

### Mice

C57BL/6 J wild-type (WT) mice were purchased from Vitonglihua Biotechnology Co., Ltd. CD169-DTR mice were kindly provided by the Laboratory of Immune Regulation, School of Life Sciences, Tokyo University of Pharmacy and Life Sciences, Tokyo, Japan, and were maintained on both C57BL/6 J and BALB/c genetic backgrounds for different experimental models. CD169-DTR mice enable DT-mediated depletion of Siglec1-expressing cells. Therefore, this model should be considered a systemic CD169⁺ cell depletion model rather than a macrophage-specific or intestine-restricted genetic targeting system. C57BL/6 J-background CD169-DTR mice were used for the azoxymethane/dextran sulfate sodium (AOM/DSS)-induced colitis-associated colorectal cancer model, whereas BALB/c-background CD169-DTR mice were used for CT26 subcutaneous tumor experiments. Male mice aged 6–8 weeks were used for all experiments unless otherwise indicated. All mice were housed under specific pathogen-free (SPF) conditions at the Animal Center of Shandong University under controlled temperature and a 12 h light–dark cycle. All animal experiments were approved by the Institutional Animal Care and Use Committee (IACUC) of Shandong University and conducted in accordance with institutional guidelines.

### AOM/DSS-induced colitis-associated colorectal cancer model

Colitis-associated colorectal cancer (CAC) was induced as previously described with minor modifications. Briefly, mice received a single intraperitoneal injection of azoxymethane (AOM, 10 mg/kg body weight). Five days later, mice were administered dextran sulfate sodium (DSS, 2–2.5%, MP Biomedicals) in drinking water for 5 consecutive days, followed by regular water for 14 days. This DSS cycle was repeated for a total of three cycles. Mice were monitored daily for body weight, clinical signs, and survival. At indicated time points (day 0, 9, 30, 60, and 80), mice were sacrificed for tissue collection. Colon length was measured immediately after sacrifice. Tumor number and size were recorded by two independent investigators blinded to group allocation.

### Diphtheria toxin-mediated depletion of CD169⁺ macrophages

For selective depletion of CD169⁺ macrophages, CD169-DTR mice received intraperitoneal injections of diphtheria toxin (DT, Sigma-Aldrich) at 25 ng/g body weight every other day starting one day before DSS administration and continuing throughout the experiment. WT littermates receiving DT served as controls.

### C3G treatment

Cyanidin-3-O-glucoside (C3G) was generously provided by Jinan University (Guangzhou, China) and confirmed to be of ≥ 98% purity. For in vivo experiments, C3G was dissolved in sterile PBS and administered intraperitoneally at the indicated dose and schedule as described for each experiment. Control mice received vehicle alone. For dose optimization experiments, cells were treated with increasing concentrations of C3G, and 4 μM was selected for subsequent in vitro experiments based on optimal efficacy.

### CT26 subcutaneous tumor model

CT26 murine colon carcinoma cells were cultured in RPMI-1640 medium supplemented with 10% fetal bovine serum (FBS) and 1% penicillin–streptomycin. For tumor implantation, 5 × 10^5^ CT26 cells were injected subcutaneously into the right flank of BALB/c-background CD169-DTR mice. Tumor size was measured every 2–3 days using calipers, and tumor volume was calculated as (length × width^2^)/2. Mice were treated with C3G or vehicle as described above. Tumors were harvested at endpoint for flow cytometry, histology, and RNA analysis.

### Human samples

Human colitis-associated colorectal cancer (CAC) specimens were obtained from the archival pathology tissue repository of Qilu Hospital, Shandong University. All cases had a documented history of ulcerative colitis and histologically confirmed colorectal cancer. The use of human samples was approved by the institutional ethics committee of Qilu Hospital, and all procedures were conducted in accordance with institutional guidelines.

### Human tissue validation analyses

For human tissue validation, paired tumor and para-tumor specimens were collected from 9 patients with colitis-associated colorectal cancer (CAC). Tumor and para-tumor regions were independently identified and confirmed by the Department of Pathology at Qilu Hospital based on histopathological evaluation. Para-tumor tissues were defined as histologically confirmed non-tumor tissues immediately adjacent to tumor lesions. Representative H&E images illustrating tumor and para-tumor regions are provided in Figure S3A. For CD169 immunohistochemical quantification, two sections per sample were analyzed, and five representative high-power fields (HPFs) were randomly selected from each section. CD169-positive cells were manually counted in each field, and the average number of positive cells per field was calculated for each sample. Quantification was performed in a blinded manner by investigators who were unaware of sample grouping. For flow cytometry analysis, freshly isolated paired tumor and para-tumor tissues were mechanically dissociated into single-cell suspensions and stained with indicated antibodies for analysis. For quantitative PCR analysis, RNA was extracted from paired tumor and para-tumor tissues and Siglec1 expression was measured using standard qPCR protocols.

### Histology and immunohistochemistry

Colon and tumor tissues from mice, as well as human colitis-associated colorectal cancer (CAC) specimens, were fixed in 10% neutral-buffered formalin, embedded in paraffin, and sectioned at 4 μm thickness. Hematoxylin and eosin (H&E) staining was performed following standard protocols. For immunohistochemistry, sections were subjected to antigen retrieval using citrate buffer (pH 6.0), blocked with 5% bovine serum albumin (BSA), and incubated overnight at 4 °C with primary antibodies against CD169 (Abcam). After washing, sections were incubated with horseradish peroxidase (HRP)–conjugated secondary antibodies, and staining was visualized using 3,3′-diaminobenzidine (DAB) substrate. Images were acquired using a light microscope.

### Immunofluorescence staining

Paraffin-embedded sections from tumor and para-tumor regions were deparaffinized and rehydrated. After antigen retrieval and blocking, sections were incubated with anti-CD169 primary antibody, followed by fluorophore-conjugated secondary antibodies. Nuclei were counterstained with DAPI. Images were acquired using a confocal microscope (Zeiss, LSM 880).

### Flow cytometry

Single-cell suspensions from colon or tumor tissues were prepared by enzymatic digestion using collagenase IV and DNase I. Cells were filtered through a 70 μm strainer and stained with fluorophore-conjugated antibodies against CD45, CD11b, CD169, CD4, CD8, and other markers as indicated. Dead cells were excluded using 7-AAD staining. Data were acquired on a BD LSRFortessa and analyzed using FlowJo software. Absolute numbers of total CD45⁺CD11b⁺ cells and CD11b⁺CD169⁺ cells were calculated based on flow cytometry data using the total number of recovered viable cells after tissue dissociation. Briefly, cells were sequentially gated for singlets, live cells, CD45⁺ immune cells, CD11b⁺ cells, and CD169⁺ cells where indicated. Absolute cell numbers were estimated by multiplying the total recovered viable cell number by the corresponding gated frequencies. For the AOM/DSS model, absolute cell numbers were normalized to colon length and reported as cells per centimeter of colon. For the CT26 tumor model, absolute cell numbers were normalized to tumor weight and reported as cells per milligram of tumor tissue. Because CD45⁺CD11b⁺ gating encompasses multiple myeloid populations, including macrophages, monocytes, neutrophils, and other CD11b⁺ cells, these measurements were interpreted as total myeloid cell abundance rather than macrophage-specific counts.

### Flow cytometric analysis of immune composition following C3G treatment

For immune composition analysis following C3G treatment, WT C57BL/6 J mice were administered intraperitoneal injections of C3G (4 μM, 200 μL per mouse) or PBS every 2 days for 7 days. Colonic tissues were collected 24 h after the final injection, digested into single-cell suspensions, and subjected to flow cytometric analysis. Cells were stained with antibodies against CD45, CD11b, F4/80, Ly6C, Ly6G, CD11c, MHC II, CD3, CD4, CD8, B220, and CD19. Dead cells were excluded using 7-AAD staining. After gating on live CD45⁺ immune cells, macrophages were identified as CD11b⁺F4/80⁺ cells, monocytes as Ly6C⁺Ly6G⁻ cells, neutrophils as Ly6G⁺Ly6C⁻ cells, dendritic cells as CD11c⁺MHC II⁺ cells, B cells as B220⁺CD19⁺ cells, and T cell subsets were identified as CD3⁺CD4⁺ or CD3⁺CD8⁺ cells.

### Isolation of CD45⁺ cells and single-cell RNA sequencing

Colon tissues were minced and digested enzymatically. CD45⁺ immune cells were enriched by fluorescence-activated cell sorting (FACS) from colon tissues and directly subjected to single-cell RNA sequencing. No additional sorting of CD169⁺ populations was performed prior to sequencing. Viable CD45⁺ cells were loaded onto the 10 × Genomics Chromium platform for single-cell RNA sequencing following the manufacturer’s protocol. Libraries were sequenced on an Illumina NovaSeq 6000 system.

### Single-cell RNA-seq data processing and analysis

Raw sequencing data were processed using Cell Ranger (v6.1). Downstream analyses were performed using Seurat (v4.3). Cells with low gene counts or high mitochondrial gene percentages were excluded. Batch effects were corrected using Harmony (v0.1). Clustering was performed using the Louvain algorithm, and clusters were annotated based on canonical marker genes. Differentially expressed genes were identified using the Wilcoxon rank-sum test.

### Definition of CD169-associated macrophage states

Macrophage populations were extracted from the integrated single-cell dataset and further subjected to unsupervised re-clustering analysis. Five transcriptionally distinct macrophage subclusters (C1–C5) were identified based on shared nearest neighbor clustering. CD169-associated macrophage states were annotated according to the relative distribution of normalized Siglec1 expression across these subclusters, together with the expression of additional functional markers. No manually predefined expression cutoffs were used for individual-cell classification. Instead, subclusters were ranked based on their average normalized Siglec1 expression levels, with C2 annotated as CD169-low, C1 annotated as CD169-mid, and C3 annotated as CD169-high. C4 and C5 were retained as additional macrophage functional states and were not included in the CD169 gradient classification. These annotation criteria were applied globally across the integrated dataset. Single-cell libraries were generated from pooled colonic tissues collected from multiple biological replicates.

### Pseudotime and trajectory analysis

Pseudotime trajectories were constructed using Monocle (v2). Macrophage subsets were ordered along inferred trajectories based on transcriptional similarity. Gene expression dynamics along pseudotime were visualized using heatmaps and smoothed expression curves.

### Cell–cell communication analysis

Cell–cell communication analysis was performed using CellPhoneDB. Ligand–receptor interaction probabilities were inferred based on curated databases. Communication networks were visualized and compared across macrophage subsets.

### Macrophage isolation and transwell co-culture assay

Paratumoral colon tissues were collected from CAC-bearing mice, as our single-cell analysis demonstrated that this region was enriched for CD169-mid and CD169-high macrophage subsets with immunosuppressive features. Tissues were mechanically dissociated and enzymatically digested into single-cell suspensions using collagenase IV and DNase I at 37 °C for 30 min. After filtration through a 70-μm cell strainer, cells were washed and resuspended in staining buffer. For flow cytometric sorting, cells were stained with fluorochrome-conjugated antibodies against CD45, CD11b, and CD169. Dead cells were excluded using 7-AAD staining. CD11b⁺CD169⁺ macrophages and CD11b⁺CD169⁻ macrophages were sorted using a FACS Aria III cell sorter (BD Biosciences). For transwell co-culture assays, CD11b⁺CD169⁺ macrophages were seeded in the upper chamber of a 0.4-μm transwell insert, while CD11b⁺CD169⁻ macrophages were seeded in the lower chamber. Cells were co-cultured for 48 h in complete RPMI-1640 medium. For blocking experiments, anti-APP neutralizing antibody or anti-CD74 blocking antibody was added to the culture system as indicated. After co-culture, CD11b⁺CD169⁻ macrophages from the lower chamber were collected for downstream RNA extraction and quantitative PCR analysis of immunosuppressive genes, including Tgfb1, Arg1, and Il10.

### Gene set enrichment analysis

Gene set enrichment analysis (GSEA) was performed using the clusterProfiler package. Hallmark and Gene Ontology (GO) gene sets were obtained from MSigDB. Enrichment scores were calculated based on differentially expressed genes or pseudotime-associated genes.

### Quantitative real-time PCR

Total RNA was extracted from tissues or cultured cells using TRIzol reagent (Invitrogen) according to the manufacturer’s instructions. First-strand cDNA was synthesized using the ReverTra Ace® qPCR RT Kit (TOYOBO). Quantitative real-time PCR was performed using THUNDERBIRD® SYBR® qPCR Mix (TOYOBO) on a QuantStudio™ 6 Flex Real-Time PCR System. Relative gene expression levels were calculated using the 2^–ΔΔCt^ method and normalized to *Gapdh*.

### In vitro tumor cell assays

CT26 cells were treated with C3G at indicated concentrations. Cell viability was assessed using CCK-8 assays. Apoptosis was analyzed using Annexin V/PI staining followed by flow cytometry.

### TCGA data analysis

RNA-seq data from TCGA COAD and READ cohorts were downloaded from the Genomic Data Commons (GDC). Normalized expression values of SIGLEC1 were compared between tumor and adjacent normal tissues.

### Statistical analysis

Statistical analysis was performed using R or GraphPad Prism. Data are presented as mean ± SD. Statistical significance was determined using Student’s t-test, two-way ANOVA, or non-parametric tests as appropriate. *P* < 0.05 was considered statistically significant.

## Supplementary Information

Below is the link to the electronic supplementary material.

Supplementary file1 Fig.S1 Flow cytometric gating strategy and validation of CD169⁺ macrophage depletion in vivo. (A) Representative flow cytometry gating strategy for identifying CD11b⁺CD169⁺ macrophages from colonic tissues, including sequential gating of live CD45⁺ immune cells. (B) Absolute quantification of macrophages and CD169⁺ macrophages in colons from AOM/DSS-treated mice and in tumors from the CT26 subcutaneous tumor model following PBS or diphtheria toxin (DT) treatment, demonstrating efficient depletion of CD169⁺ macrophages. Data are presented as mean ± SD. ****P < 0.0001 (unpaired Student’s t-test) (PDF 1649 KB)

Supplementary file2 Fig.S2 CD169-associated macrophage states and transcriptional features. (A) UMAP visualization of macrophage subsets colored by CD169 expression states, including CD169-high, CD169-mid, CD169-low, and CD169-neg populations. (B) Violin plots showing M1 and M2 gene program scores across CD169-high, CD169-mid, and CD169-low macrophage subsets. Statistical significance was determined using unpaired Student’s t-test. (C) Network representation of enriched biological pathways based on differentially expressed genes in CD169-high macrophages, highlighting functional modules associated with immune regulation (PDF 1673 KB)

Supplementary file3 Fig.S3 Histopathological validation of para-tumor regions and flow cytometry gating strategy for human CD169⁺ cell analysis. (A) Representative H&E staining of paired human colitis-associated colorectal cancer tumor and para-tumor (Para-T) tissues. Para-tumor regions were defined as histologically confirmed non-tumor tissues immediately adjacent to tumor lesions based on pathological evaluation. Representative images from four independent patients are shown. Scale bar, 100 μm. (B) Representative flow cytometry gating strategy for identifying CD169⁺ cells from freshly isolated human colitis-associated colorectal cancer specimens. Live CD45⁺ immune cells were sequentially gated following FSC/SSC and 7-AAD exclusion, followed by identification of CD68⁺CD169⁺ cells. (C) Normalized SIGLEC1 expression in para-tumor and tumor tissues from TCGA COAD and READ cohorts (PDF 4041 KB)

Supplementary file4 Fig.S4 CD169⁺ macrophages promote immunosuppressive macrophage reprogramming through APP–CD74 signaling. (A) Schematic illustration of the in vitro transwell co-culture assay. CD11b⁺CD169⁺ and CD11b⁺CD169⁻ macrophages were isolated from paratumoral colon tissues of colitis-associated colorectal cancer (CAC) mice and subjected to indirect co-culture. Four experimental groups were included: CD169⁻ macrophages alone, CD169⁻ macrophages co-cultured with CD169⁺ macrophages, co-culture with anti-APP neutralizing antibody treatment, and co-culture with anti-CD74 blocking antibody treatment. Downstream qPCR analysis was performed in CD169⁻ macrophages collected from the lower chamber. (B) Representative flow cytometry gating strategy for isolating CD11b⁺CD169⁺ and CD11b⁺CD169⁻ macrophages from paratumoral colon tissues. (C) Quantitative PCR analysis showing increased App expression in CD169⁺ macrophages and relatively higher Cd74 expression in CD169⁻ macrophages. (D) Quantitative PCR analysis of Tgfb1, Arg1, and Il10 expression in CD169⁻ macrophages following co-culture with CD169⁺ macrophages, with or without APP/CD74 blockade. Data are presented as mean ± SD. *P < 0.05, **P < 0.01, ***P < 0.001 (PDF 2323 KB)

Supplementary file5 Fig.S5 Dose optimization and additional effects of C3G treatment. (A) Dose–response analysis of C3G treatment showing that 4 μM resulted in the maximal reduction of CD169 expression, whereas higher concentrations did not provide additional benefit. (B) Representative immunofluorescence staining of para-tumor tissues showing reduced CD169 expression following C3G treatment. (C–F) In vitro analyses demonstrating direct effects of C3G on tumor cells. C3G treatment promoted tumor cell apoptosis and reduced cell viability in a dose-dependent manner, as assessed by apoptosis assays and cell viability measurements. Data are shown as mean ± SD. Statistical significance was determined using unpaired Student’s t-test or one-way ANOVA where applicable (PDF 2636 KB)

Supplementary file6 Fig.S6 C3G selectively modulates CD169-associated macrophages without broadly altering immune cell composition. (A) Representative flow cytometry gating strategy for profiling major immune cell populations in colonic tissues following short-term C3G treatment in WT mice. Live CD45⁺ immune cells were sequentially gated to identify macrophages (CD11b⁺F4/80⁺), B cells (B220⁺CD19⁺), monocytes (Ly6C⁺Ly6G⁻), neutrophils (Ly6G⁺Ly6C⁻), dendritic cells (CD11c⁺MHC II⁺), and CD4⁺/CD8⁺ T cell populations. (B) Quantification of major immune cell populations in vehicle- and C3G-treated WT mice, showing that C3G did not significantly alter overall immune cell composition. (C) Representative gross images of colons from PBS-, DT-, C3G-, and DT+C3G-treated mice. (D) Colon length measurements across indicated treatment groups. (E) Body weight changes during disease progression in PBS-, DT-, C3G-, and DT+C3G-treated mice. Data are presented as mean ± SD. Statistical significance was determined using unpaired Student’s t-test or one-way ANOVA where appropriate. ns, not significant; *P < 0.05; **P < 0.01 (PDF 6573 KB)

## Data Availability

The single-cell RNA sequencing data generated in this study are publicly available at: https://pan.baidu.com/s/1b2l9fxRoTcbvQ72tb5TUZw?pwd=ti34. All analysis scripts used in this study are available from the corresponding author upon reasonable request.

## Material availability

This study did not generate new unique reagents.

## References

[1] Rogler G. Chronic ulcerative colitis and colorectal cancer. Cancer Lett. 2014;345(2):235–41. 10.1016/j.canlet.2013.07.032 23941831

[2] Shah SC, Itzkowitz SH. Colorectal cancer in inflammatory bowel disease: mechanisms and management. Gastroenterology. 2022;162(3):715-30.e3. 10.1053/j.gastro.2021.10.035 PMC9003896 34757143

[3] Nardone OM, Zammarchi I, Santacroce G, Ghosh S, Iacucci M. Inflammation-driven colorectal cancer associated with colitis: from pathogenesis to changing therapy. Cancers (Basel). 2023. 10.3390/cancers15082389. PMC10136846 37190315

[4] Pandey A, Shen C, Man SM. Inflammasomes in colitis and colorectal cancer: mechanism of action and therapies. Yale J Biol Med. 2019;92(3):481–98. PMC6747943 31543710

[5] Dong Y, Yang Q, Niu R, Zhang Z, Huang Y, Bi Y, et al. Modulation of tumor-associated macrophages in colitis-associated colorectal cancer. J Cell Physiol. 2022;237(12):4443–59. 10.1002/jcp.30906 36302153

[6] Fantini MC, Favale A, Onali S, Facciotti F. Tumor infiltrating regulatory T cells in sporadic and colitis-associated colorectal cancer: the red little riding hood and the wolf. Int J Mol Sci. 2020. 10.3390/ijms21186744. PMC7555219 32937953

[7] Filardy AA, Ferreira JRM, Rezende RM, Kelsall BL, Oliveira RP. The intestinal microenvironment shapes macrophage and dendritic cell identity and function. Immunol Lett. 2023;253:41–53. 10.1016/j.imlet.2023.01.003 PMC9907447 36623708

[8] Leman JKH, Munoz-Erazo L, Kemp RA. The intestinal tumour microenvironment. Adv Exp Med Biol. 2020;1226:1–22. 10.1007/978-3-030-36214-0_1 32030672

[9] Xu R, Vujić N, Bianco V, Reinisch I, Kratky D, Krstic J, et al. Lipid-associated macrophages between aggravation and alleviation of metabolic diseases. Trends Endocrinol Metab. 2024;35(11):981–95. 10.1016/j.tem.2024.04.009 38705759

[10] Jin R, Neufeld L, McGaha TL. Linking macrophage metabolism to function in the tumor microenvironment. Nat Cancer. 2025;6(2):239–52. 10.1038/s43018-025-00909-2 39962208

[11] Wynn TA, Vannella KM. Macrophages in tissue repair, regeneration, and fibrosis. Immunity. 2016;44(3):450–62. 10.1016/j.immuni.2016.02.015 PMC4794754 26982353

[12] Lazarov T, Juarez-Carreño S, Cox N, Geissmann F. Physiology and diseases of tissue-resident macrophages. Nature. 2023;618(7966):698–707. 10.1038/s41586-023-06002-x PMC10649266 37344646

[13] Chen R, Zhang H, Tang B, Luo Y, Yang Y, Zhong X, et al. Macrophages in cardiovascular diseases: molecular mechanisms and therapeutic targets. Signal Transduct Target Ther. 2024;9(1):130. 10.1038/s41392-024-01840-1 PMC11139930 38816371

[14] Mass E, Nimmerjahn F, Kierdorf K, Schlitzer A. Tissue-specific macrophages: how they develop and choreograph tissue biology. Nat Rev Immunol. 2023;23(9):563–79. 10.1038/s41577-023-00848-y PMC10017071 36922638

[15] Asano K, Kikuchi K, Tanaka M. CD169 macrophages regulate immune responses toward particulate materials in the circulating fluid. J Biochem. 2018;164(2):77–85. 10.1093/jb/mvy050 29905851

[16] Asano K, Nabeyama A, Miyake Y, Qiu CH, Kurita A, Tomura M, et al. CD169-positive macrophages dominate antitumor immunity by crosspresenting dead cell-associated antigens. Immunity. 2011;34(1):85–95. 10.1016/j.immuni.2010.12.011 21194983

[17] Asano K, Takahashi N, Ushiki M, Monya M, Aihara F, Kuboki E, et al. Intestinal CD169(+) macrophages initiate mucosal inflammation by secreting CCL8 that recruits inflammatory monocytes. Nat Commun. 2015;6:7802. 10.1038/ncomms8802 PMC4518321 26193821

[18] Jing W, Guo X, Wang G, Bi Y, Han L, Zhu Q, et al. Breast cancer cells promote CD169(+) macrophage-associated immunosuppression through JAK2-mediated PD-L1 upregulation on macrophages. Int Immunopharmacol. 2020;78:106012. 10.1016/j.intimp.2019.106012 31865052

[19] Song X, Li N, Liu Y, Wang Z, Wang T, Tan S, et al. CD169-positive macrophages enhance abscopal effect of radiofrequency ablation therapy in liver cancer. Transl Oncol. 2022;15(1):101306. 10.1016/j.tranon.2021.101306 PMC8662336 34883446

[20] Komohara Y, Ohnishi K, Takeya M. Possible functions of CD169-positive sinus macrophages in lymph nodes in anti-tumor immune responses. Cancer Sci. 2017;108(3):290–5. 10.1111/cas.13137 PMC5378284 28002629

[21] Bouma RG, Wang AZ, den Haan JMM. Exploring CD169(+) macrophages as key targets for vaccination and therapeutic interventions. Vaccines. 2025. 10.3390/vaccines13030330. PMC11946325 40266235

[22] Liu Y, Xia Y, Qiu CH. Functions of CD169 positive macrophages in human diseases (Review). Biomed Rep. 2021;14(2):26. 10.3892/br.2020.1402 PMC7780751 33408860

[23] Dzhalilova D, Zolotova N, Fokichev N, Makarova O. Murine models of colorectal cancer: the azoxymethane (AOM)/dextran sulfate sodium (DSS) model of colitis-associated cancer. PeerJ. 2023;11:e16159. 10.7717/peerj.16159 PMC10624171 37927787

[24] Zhao JW, Zhao WY, Yu ZY. The roles of dietary bioactive compounds in alleviating inflammatory bowel disease-associated colorectal cancer. Food Sci Nutr. 2025;13(6):e70432. 10.1002/fsn3.70432 PMC12179676 40548181

[25] Xia Y, Tian LM, Liu Y, Guo KS, Lv M, Li QT, et al. Low dose of cyanidin-3-O-glucoside alleviated dextran sulfate sodium-induced colitis, mediated by CD169+ macrophage pathway. Inflamm Bowel Dis. 2019;25(9):1510–21. 10.1093/ibd/izz090 31107535

[26] Martinez-Pomares L, Gordon S. CD169+ macrophages at the crossroads of antigen presentation. Trends Immunol. 2012;33(2):66–70. 10.1016/j.it.2011.11.001 22192781

[27] Herzog S, Fragkou PC, Arneth BM, Mkhlof S, Skevaki C. Myeloid CD169/Siglec1: an immunoregulatory biomarker in viral disease. Front Med. 2022;9:979373. 10.3389/fmed.2022.979373 PMC9540380 36213653

[28] Eakin AJ, Bustard MJ, McGeough CM, Ahmed T, Bjourson AJ, Gibson DS. Siglec-1 and -2 as potential biomarkers in autoimmune disease. Proteomics Clin Appl. 2016;10(6):635–44. 10.1002/prca.201500069 26752092

[29] Li Q, Wang D, Hao S, Han X, Xia Y, Li X, et al. CD169 expressing macrophage, a key subset in mesenteric lymph nodes promotes mucosal inflammation in dextran sulfate sodium-induced colitis. Front Immunol. 2017;8:669. 10.3389/fimmu.2017.00669 PMC5483437 28694804

[30] Wang D, Li Q, Yang Y, Hao S, Han X, Song J, et al. Macrophage subset expressing CD169 in peritoneal cavity-regulated mucosal inflammation together with lower levels of CCL22. Inflammation. 2017;40(4):1191–203. 10.1007/s10753-017-0562-0 28466432

[31] Hu J, Guo F, Han C, Zhao Q, Yi H, Xu J, et al. CD169(+) macrophages identify and eliminate tumor cells in colorectal cancer through CD169/CD43 interaction and FasL-driven apoptosis. Cell Rep. 2025;44(10):116351. 10.1016/j.celrep.2025.116351 41016034

[32] Saito Y, Fujiwara Y, Miyamoto Y, Ohnishi K, Nakashima Y, Tabata Y, et al. CD169(+) sinus macrophages in regional lymph nodes do not predict mismatch-repair status of patients with colorectal cancer. Cancer Med. 2023;12(9):10199–211. 10.1002/cam4.5747 PMC10225197 36846928

[33] Ohnishi K, Komohara Y, Saito Y, Miyamoto Y, Watanabe M, Baba H, et al. CD169-positive macrophages in regional lymph nodes are associated with a favorable prognosis in patients with colorectal carcinoma. Cancer Sci. 2013;104(9):1237–44. 10.1111/cas.12212 PMC7657174 23734742

[34] Shi CL, Han XL, Chen JC, Pan QF, Gao YC, Guo PY, et al. Single-nucleus transcriptome unveils the role of ferroptosis in ischemic stroke. Heliyon. 2024;10(12):e32727. 10.1016/j.heliyon.2024.e32727 PMC11237950 38994078

[35] Wang T, Hao J, Li B, Hyraht A, Wang J, Xia H, et al. Single-cell and spatial transcriptomics reveal a stress-induced EMT-like epithelial subset driving immune activation in silica-injured lung. Front Immunol. 2025;16:1609616. 10.3389/fimmu.2025.1609616 PMC12179088 40547038

[36] Gan Y, Fu Y, Yang L, Chen J, Lei H, Liu Q. Cyanidin-3-O-glucoside and cyanidin protect against intestinal barrier damage and 2,4,6-Trinitrobenzenesulfonic acid-induced colitis. J Med Food. 2020;23(1):90–9. 10.1089/jmf.2019.4524 31657664

[37] Oumeddour DZ, Al-Dalali S, Zhao L, Zhao L, Wang C. Recent advances on cyanidin-3-O-glucoside in preventing obesity-related metabolic disorders: a comprehensive review. Biochem Biophys Res Commun. 2024;729:150344. 10.1016/j.bbrc.2024.150344 38976946

[38] Miyake Y, Asano K, Kaise H, Uemura M, Nakayama M, Tanaka M. Critical role of macrophages in the marginal zone in the suppression of immune responses to apoptotic cell-associated antigens. J Clin Invest. 2007;117(8):2268–78. 10.1172/JCI31990 PMC1924497 17657313

