# Supplementary material for: Stage-associated remodeling of CD169 macrophage states is linked to immune regulation in colitis-associated colorectal cancer
Source: J Gastroenterol. 2026 Jun 24;61(11):1563–81. doi: 10.1007/s00535-026-02463-9 (PMC13695605; doi:10.1007/s00535-026-02463-9)
Supplement: Supplementary file 1 [file 535_2026_2463_MOESM1_ESM.pdf]

Figure S1

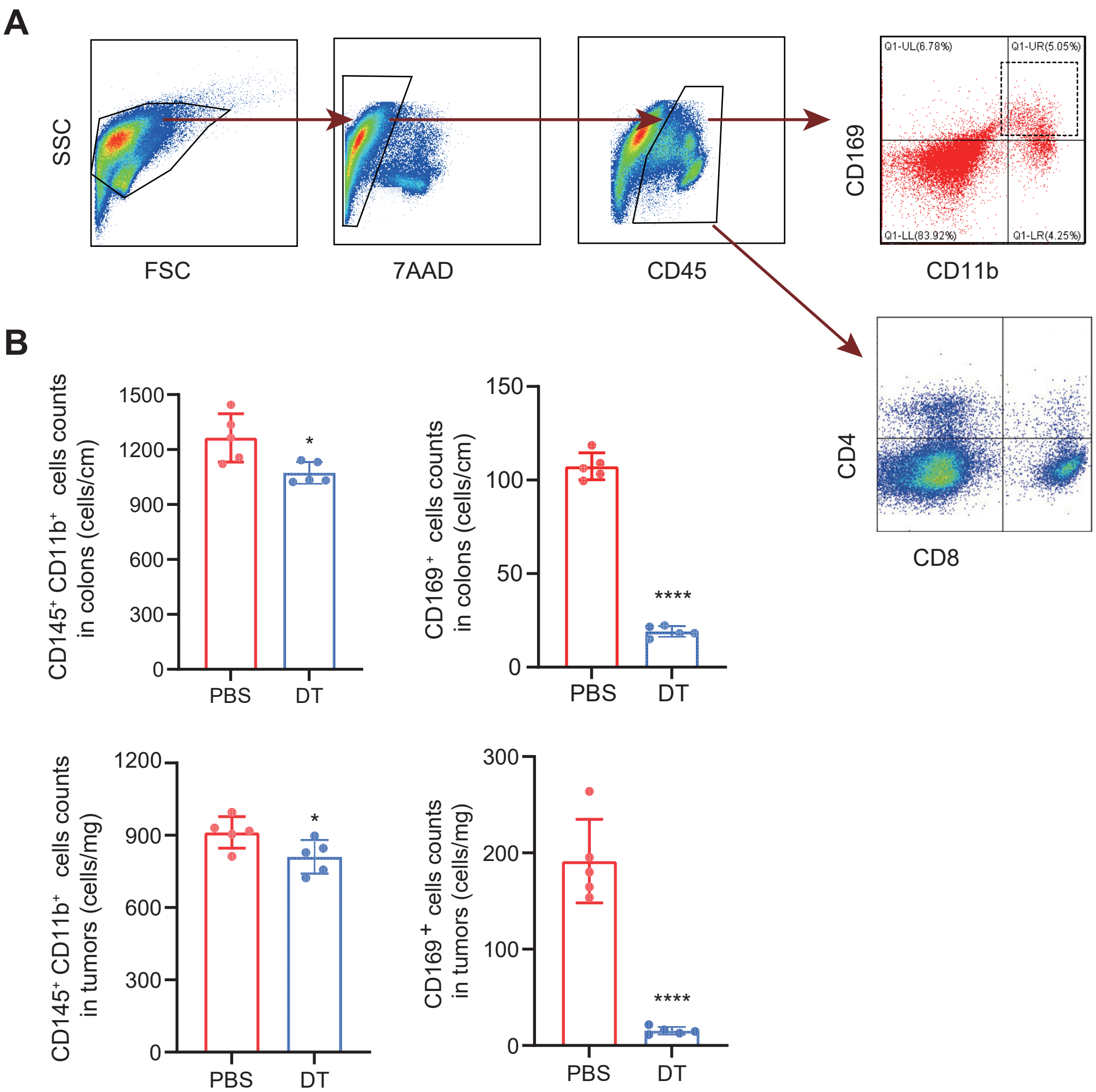

A
SSC
FSC
7AAD
CD45
CD169
CD11b
Q1-UL(6.78%)
Q1-UR(5.05%)
Q1-LL(83.92%)
Q1-LR(4.25%)
CD4
CD8
B
CD145+ CD11b+ cells counts in colons (cells/cm)
1500
1200
900
600
300
0
*
PBS
DT
CD169+ cells counts in colons (cells/cm)
150
100
50
0
****
PBS
DT
CD145+ CD11b+ cells counts in tumors (cells/mg)
1200
900
600
300
0
*
PBS
DT
CD169+ cells counts in tumors (cells/mg)
300
200
100
0
****
PBS
DT
