# Supplementary material for: Stage-associated remodeling of CD169 macrophage states is linked to immune regulation in colitis-associated colorectal cancer
Source: J Gastroenterol. 2026 Jun 24;61(11):1563–81. doi: 10.1007/s00535-026-02463-9 (PMC13695605; doi:10.1007/s00535-026-02463-9)
Supplement: Supplementary file 2 [file 535_2026_2463_MOESM2_ESM.pdf]

Figure S2

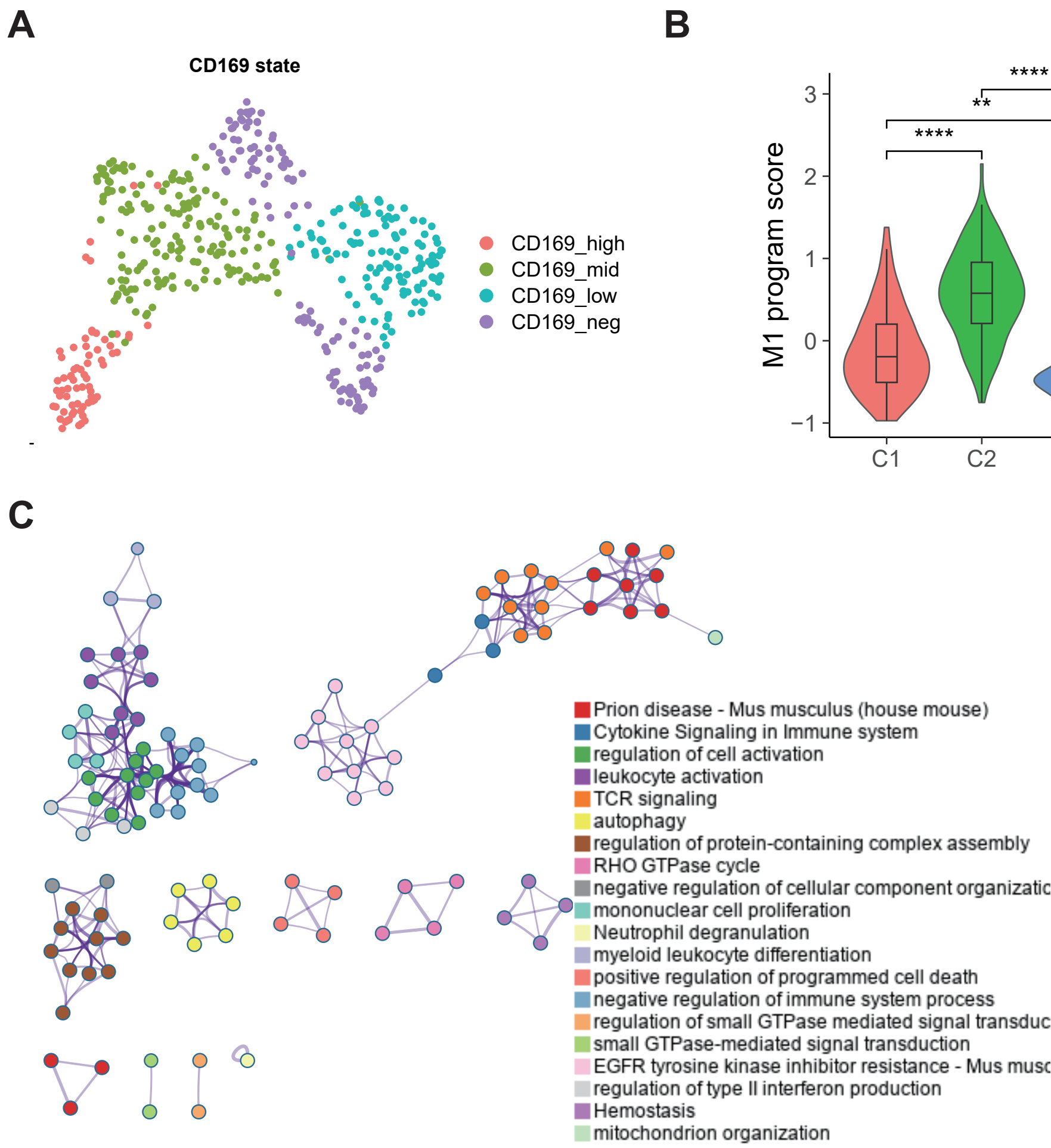
A
CD169 state
CD169_high
CD169_mid
CD169_low
CD169_neg
B
M1 program score
M2 program score
C1
C2
C3
****
**
***
C
Prion disease - Mus musculus (house mouse)
Cytokine Signaling in Immune system
regulation of cell activation
leukocyte activation
TCR signaling
autophagy
regulation of protein-containing complex assembly
RHO GTPase cycle
negative regulation of cellular component organizatic
mononuclear cell proliferation
Neutrophil degranulation
myeloid leukocyte differentiation
positive regulation of programmed cell death
negative regulation of immune system process
regulation of small GTPase mediated signal transduc
small GTPase-mediated signal transduction
EGFR tyrosine kinase inhibitor resistance - Mus musc
regulation of type II interferon production
Hemostasis
mitochondrion organization
