# Supplementary material for: Stage-associated remodeling of CD169 macrophage states is linked to immune regulation in colitis-associated colorectal cancer
Source: J Gastroenterol. 2026 Jun 24;61(11):1563–81. doi: 10.1007/s00535-026-02463-9 (PMC13695605; doi:10.1007/s00535-026-02463-9)

Figure S3

A

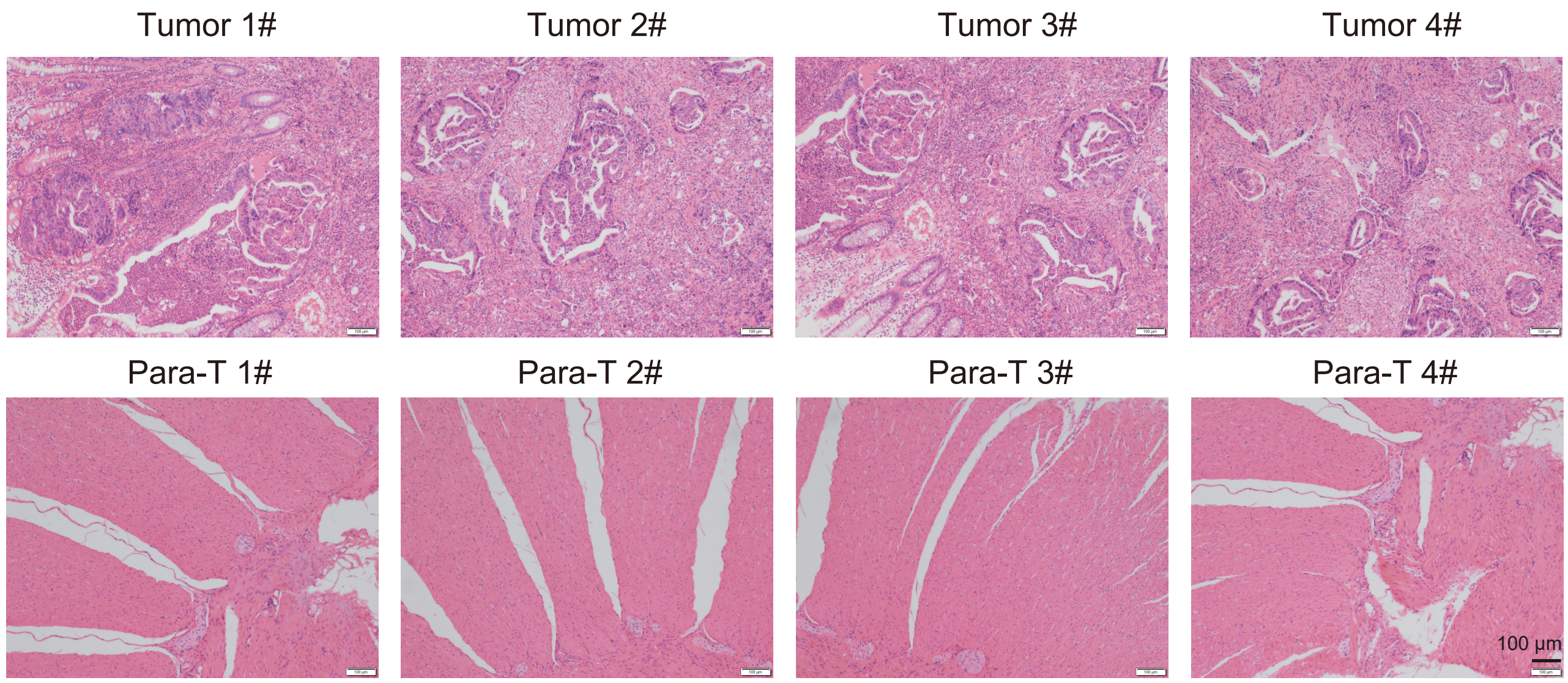

B

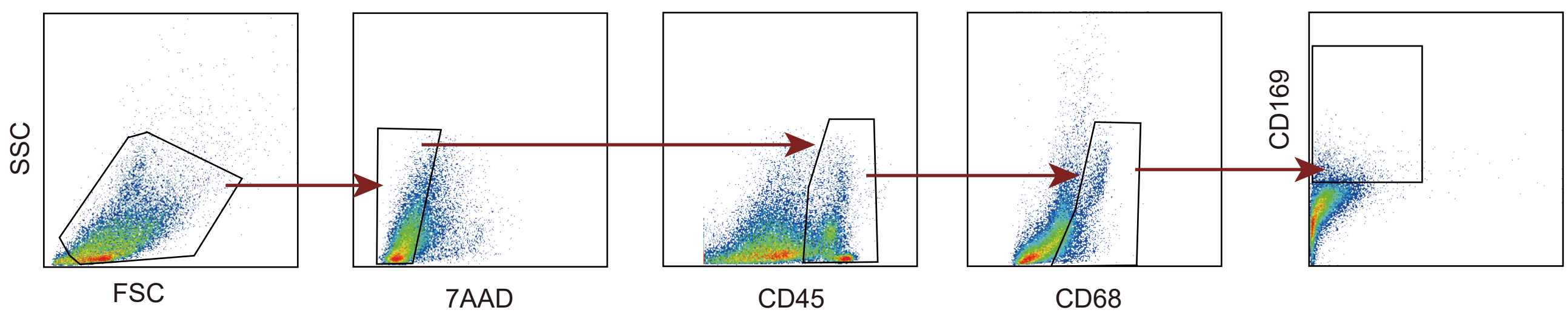

C

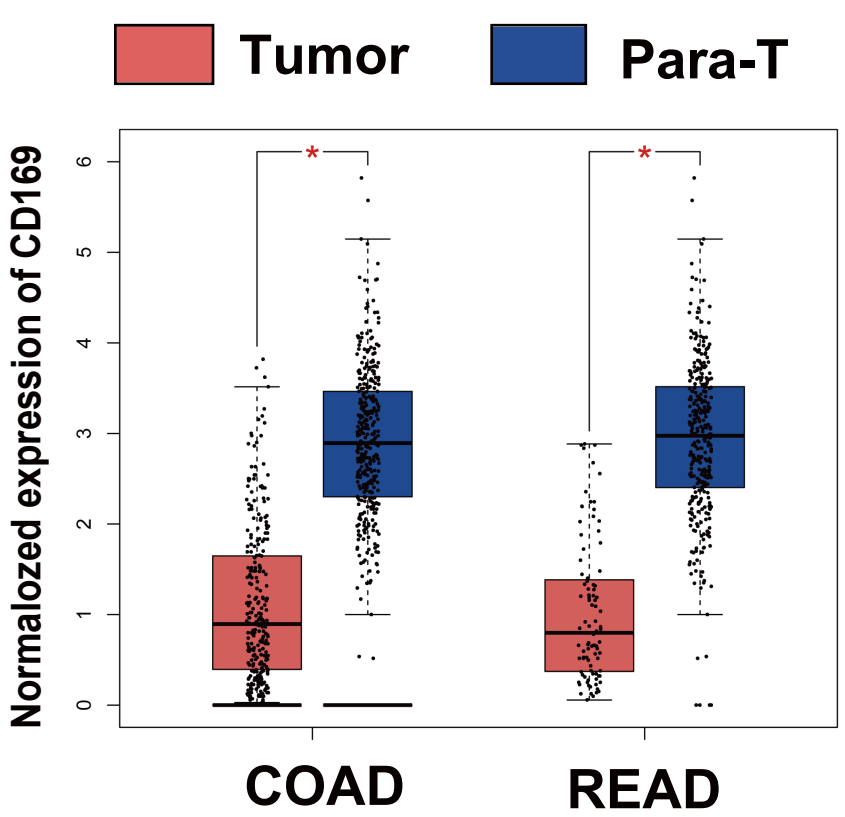

Supplement: Supplementary file 3 [file 535_2026_2463_MOESM3_ESM.pdf]
