# Supplementary material for: Stage-associated remodeling of CD169 macrophage states is linked to immune regulation in colitis-associated colorectal cancer
Source: J Gastroenterol. 2026 Jun 24;61(11):1563–81. doi: 10.1007/s00535-026-02463-9 (PMC13695605; doi:10.1007/s00535-026-02463-9)
Supplement: Supplementary file 4 [file 535_2026_2463_MOESM4_ESM.pdf]

Figure S4

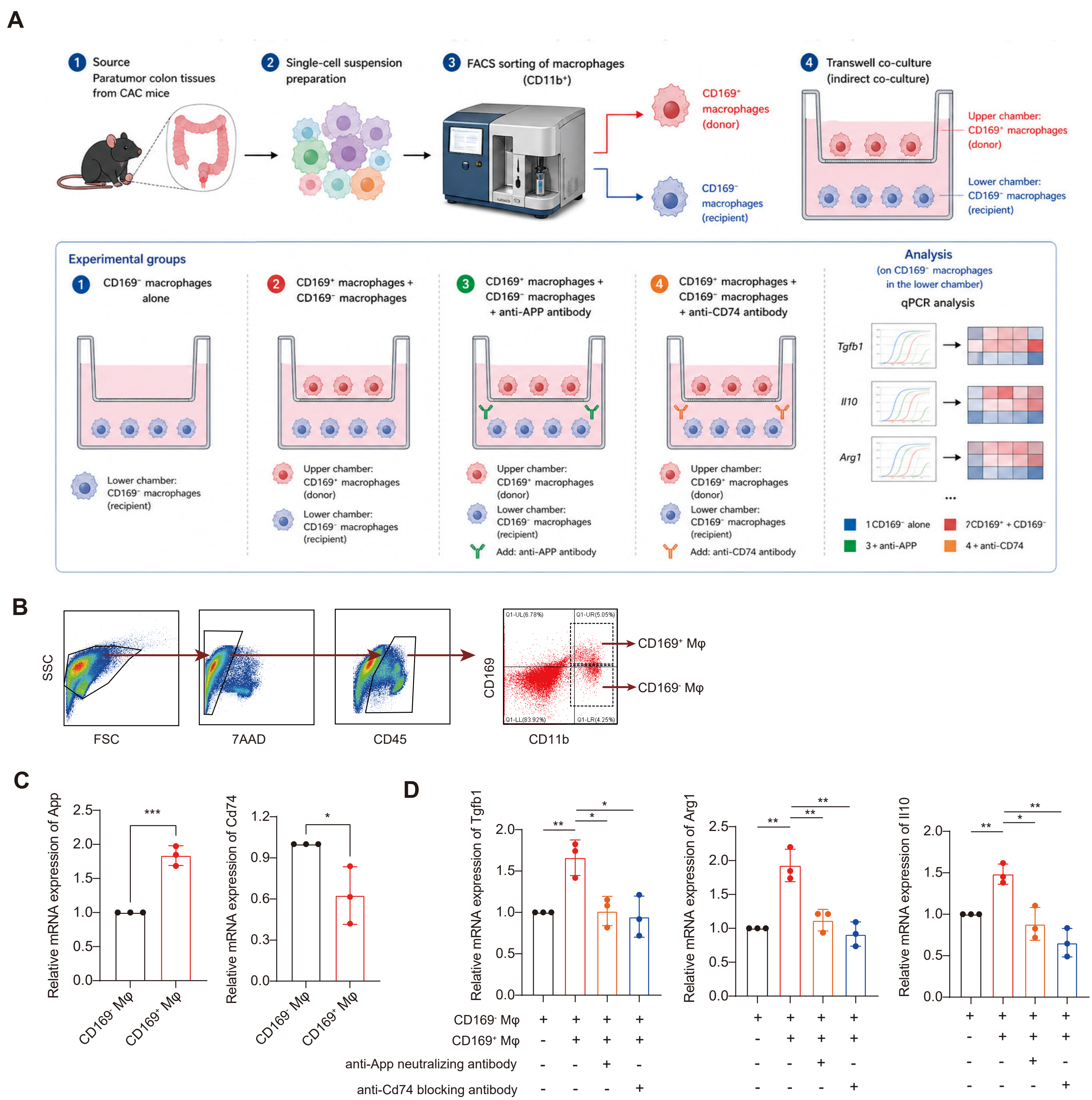
A
1 Source
Paratumor colon tissues from CAC mice
2 Single-cell suspension preparation
3 FACS sorting of macrophages (CD11b⁺)
CD169⁺ macrophages (donor)
CD169⁻ macrophages (recipient)
4 Transwell co-culture (indirect co-culture)
Upper chamber: CD169⁺ macrophages (donor)
Lower chamber: CD169⁻ macrophages (recipient)
Experimental groups
1 CD169⁻ macrophages alone
2 CD169⁺ macrophages + CD169⁻ macrophages
3 CD169⁺ macrophages + CD169⁻ macrophages + anti-APP antibody
4 CD169⁺ macrophages + CD169⁻ macrophages + anti-CD74 antibody
Lower chamber: CD169⁻ macrophages (recipient)
Upper chamber: CD169⁺ macrophages (donor)
Add: anti-APP antibody
Add: anti-CD74 antibody
Analysis (on CD169⁻ macrophages in the lower chamber)
qPCR analysis
Tgfb1
Il10
Arg1
1 CD169⁻ alone
7 CD169⁺ + CD169⁻
3 + anti-APP
4 + anti-CD74
B
SSC
FSC
7AAD
CD45
CD169
CD11b
Q1-UL(6.78%)
Q1-UR(5.05%)
Q1-LL(83.92%)
Q1-LR(4.25%)
CD169⁺ Mφ
CD169⁻ Mφ
C
Relative mRNA expression of App
Relative mRNA expression of Cd74
CD169⁻ Mφ
CD169⁺ Mφ
D
Relative mRNA expression of Tgfb1
Relative mRNA expression of Arg1
Relative mRNA expression of Il10
CD169⁻ Mφ
CD169⁺ Mφ
anti-App neutralizing antibody
anti-Cd74 blocking antibody
