# Supplementary material for: Stage-associated remodeling of CD169 macrophage states is linked to immune regulation in colitis-associated colorectal cancer
Source: J Gastroenterol. 2026 Jun 24;61(11):1563–81. doi: 10.1007/s00535-026-02463-9 (PMC13695605; doi:10.1007/s00535-026-02463-9)
Supplement: Supplementary file 5 [file 535_2026_2463_MOESM5_ESM.pdf]

Figure S5

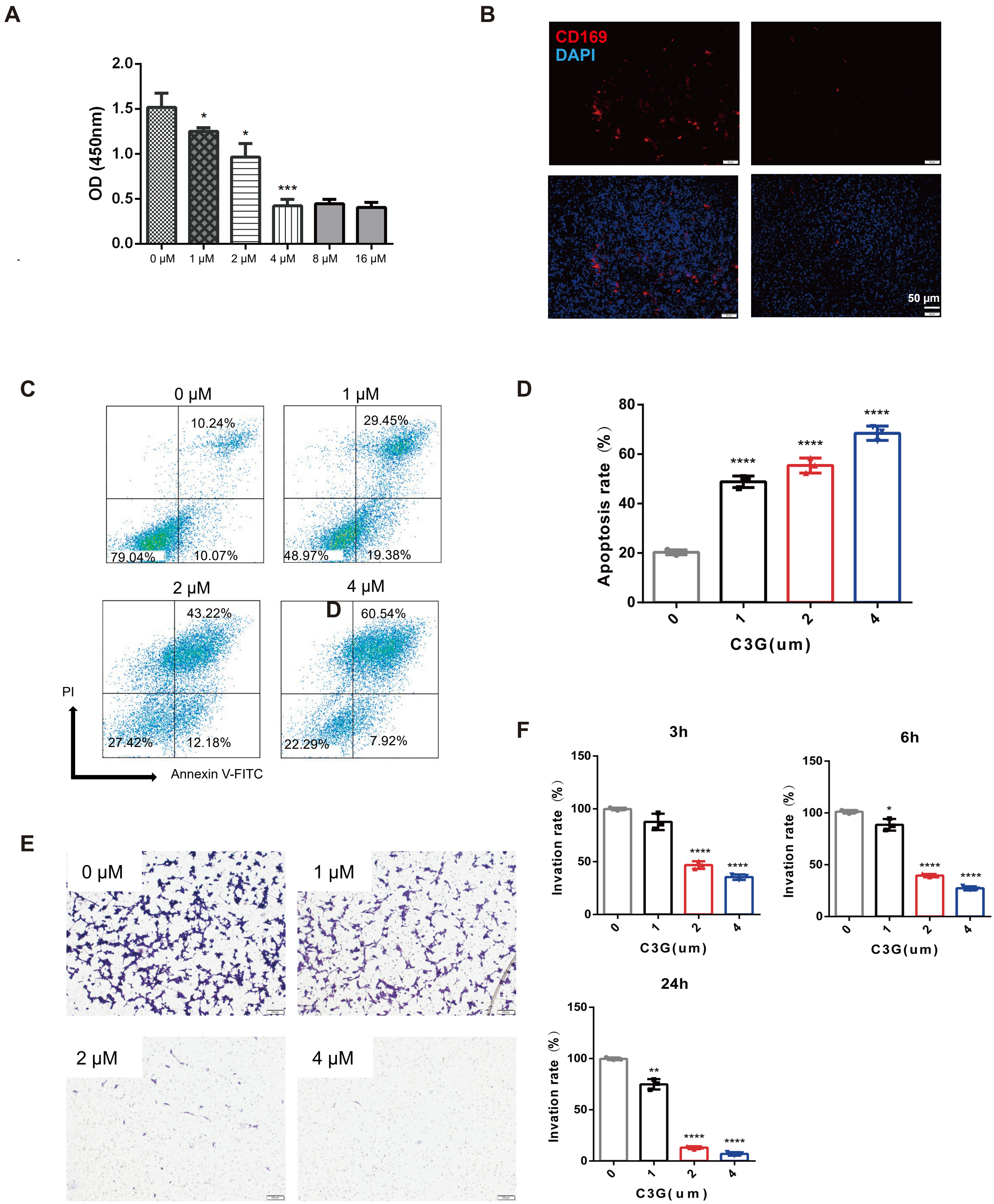
A
OD (450nm)
2.0
1.5
1.0
0.5
0.0
0 μM
1 μM
2 μM
4 μM
8 μM
16 μM
B
CD169
DAPI
50 μm
C
0 μM
10.24%
79.04%
10.07%
1 μM
29.45%
48.97%
19.38%
2 μM
43.22%
27.42%
12.18%
4 μM
60.54%
22.29%
7.92%
PI
Annexin V-FITC
D
Apoptosis rate (%)
80
60
40
20
0
C3G(um)
E
0 μM
1 μM
2 μM
4 μM
F
3h
6h
24h
Invation rate (%)
150
100
50
0
C3G(um)
