# Supplementary material for: Stage-associated remodeling of CD169 macrophage states is linked to immune regulation in colitis-associated colorectal cancer
Source: J Gastroenterol. 2026 Jun 24;61(11):1563–81. doi: 10.1007/s00535-026-02463-9 (PMC13695605; doi:10.1007/s00535-026-02463-9)
Supplement: Supplementary file 6 [file 535_2026_2463_MOESM6_ESM.pdf]

Figure S6

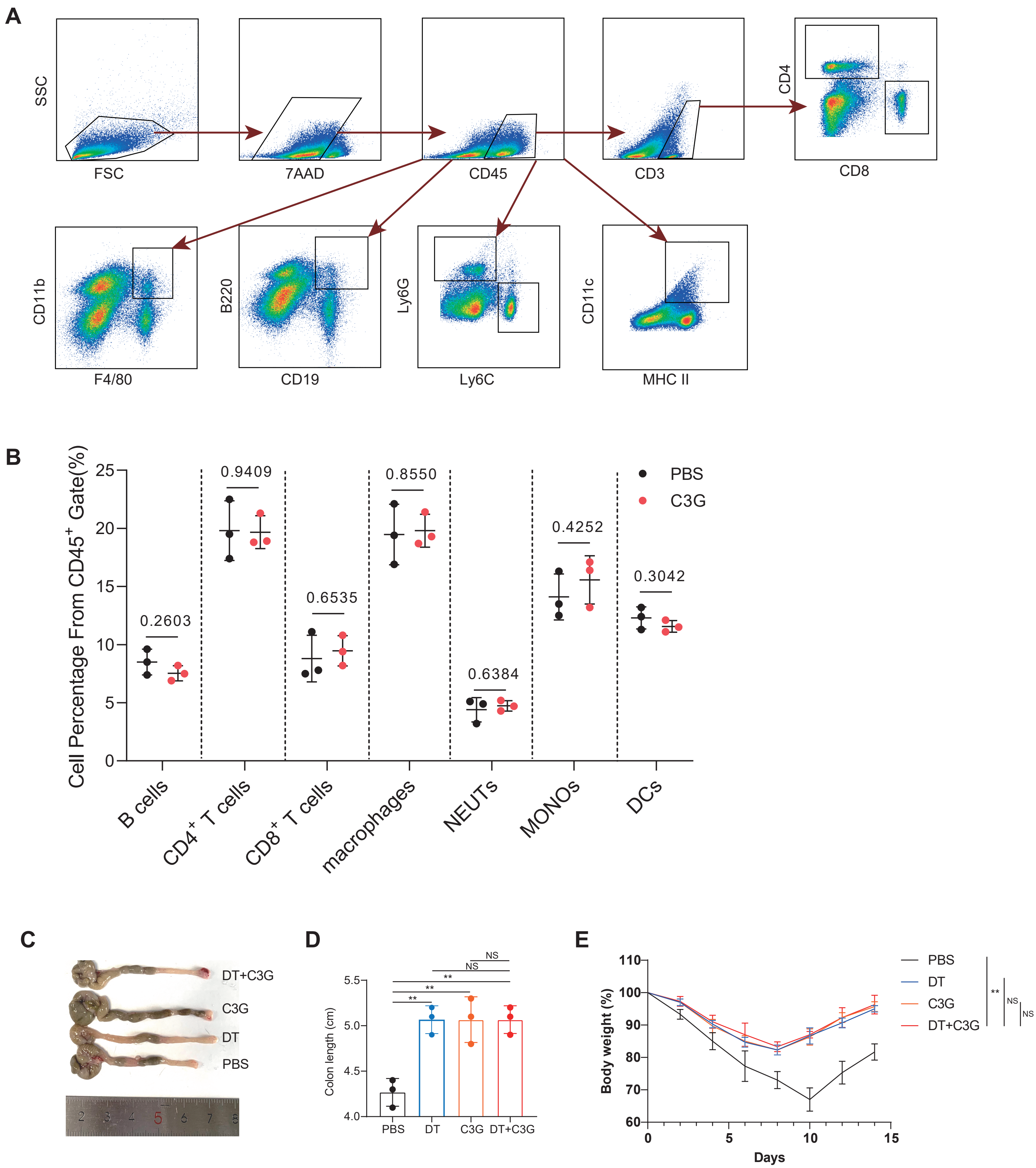

A
SSC
FSC
7AAD
CD45
CD3
CD4
CD8
CD11b
F4/80
B220
CD19
Ly6G
Ly6C
CD11c
MHC II
B
Cell Percentage From CD45+ Gate(%)
0
5
10
15
20
25
0.2603
0.9409
0.6535
0.8550
0.6384
0.4252
0.3042
PBS
C3G
B cells
CD4+ T cells
CD8+ T cells
macrophages
NEUTs
MONOs
DCs
C
DT+C3G
C3G
DT
PBS
D
Colon length (cm)
4.0
4.5
5.0
5.5
NS
NS
**
**
**
PBS
DT
C3G
DT+C3G
E
Body weight (%)
60
70
80
90
100
110
0
5
10
15
Days
PBS
DT
C3G
DT+C3G
**
NS
NS
